# Evolution of a transposon in *Daphnia* hybrid genomes

**DOI:** 10.1186/1759-8753-4-7

**Published:** 2013-02-06

**Authors:** Roland Vergilino, Tyler A Elliott, Philippe Desjardins-Proulx, Teresa J Crease, France Dufresne

**Affiliations:** 1 Département de Biologie, Université du Québec à Rimouski, 300, allée des Ursulines, G5L 3A1, Rimouski, Québec, Canada; 2 Centre d’Études Nordiques, Université Laval, Pavillon Abitibi-Price, 2405, rue de la Terrasse, Local 1202, G1V 0A6, Québec, Canada; 3 Current address: Great Lake Institute of Environmental Research, University of Windsor, 401 Sunset Avenue, N9B 3P4, Windsor, Canada; 4 Department of Integrative Biology, University of Guelph, Science complex, N1G 2 W1, Guelph, ON, Canada; 5 College of Engineering, University of Illinois at Chicago, (M/C 159), 851 South Morgan, 60607, Chicago, IL, USA

**Keywords:** *Daphnia pulex*, Transposable element, *Pokey*, Hybrids, Recombination

## Abstract

**Background:**

Transposable elements play a major role in genome evolution. Their capacity to move and/or multiply in the genome of their host may have profound impacts on phenotypes, and may have dramatic consequences on genome structure. Hybrid and polyploid clones have arisen multiple times in the *Daphnia pulex* complex and are thought to reproduce by obligate parthenogenesis. Our study examines the evolution of a DNA transposable element named *Pokey* in the *D. pulex* complex.

**Results:**

Portions of *Pokey* elements inserted in the 28S rRNA genes from various *Daphnia* hybrids (diploids and polyploids) were sequenced and compared to sequences from a previous study to understand the evolutionary history of the elements. *Pokey* sequences show a complex phylogenetic pattern. We found evidence of recombination events in numerous *Pokey* alleles from diploid and polyploid hybrids and also from non-hybrid diploids. The recombination rate in *Pokey* elements is comparable to recombination rates previously estimated for 28S rRNA genes in the congener, *Daphnia obtusa.* Some recombinant *Pokey* alleles were encountered in *Daphnia* isolates from multiple locations and habitats.

**Conclusions:**

Phylogenetic and recombination analyses showed that recombination is a major force that shapes *Pokey* evolution. Based on *Pokey* phylogenies, reticulation has played and still plays an important role in shaping the diversity of the *D. pulex* complex. Horizontal transfer of *Pokey* seems to be rare and hybrids often possess *Pokey* elements derived from recombination among alleles encountered in the putative parental species. The insertion of *Pokey* in hotspots of recombination may have important impacts on the diversity and fitness of this transposable element.

## Background

Transposable elements (TEs) are widespread in the living world. Few multicellular eukaryotes lack these genetic elements, which have the ability to move and multiply throughout the genome [1], but see [2] for exceptions in endosymbiont genomes. There is substantial variation in the proportion of mobile genetic elements and especially TEs across genomes [3]. Numerous studies have explored whether TE history reflects the evolutionary history of their hosts (that is, co-evolution [4-7]). Due to their high densities in eukaryotic genomes and their irreversible mode of insertion, some authors have proposed that SINEs (Short INterspersed Elements, non-autonomous Class I TEs) may be used for inferring the evolutionary history of their host [8]. The phylogeny of a strictly vertically transmitted TE (from parents to offspring) should reflect that of its host(s). However, genetic and evolutionary processes, which may occur during speciation, may affect subsequent phylogenetic inferences based on TE sequences. For example, horizontal transfer (that is, transmission by vectors unrelated to host reproduction), incomplete lineage sorting (that is, the maintenance of ancient polymorphism in closely related species), lack of genetic variability or hybridization/introgression may account for incongruent phylogenetic patterns between TEs and their hosts [9-13]. Hybrid genomes should carry TE(s) from the genomes of their parental species. In contrast, if horizontal transfers occur between species that share ecological niches or are infected by the same parasites, we do not expect that TE phylogenies will match species phylogenies.

The *Daphnia pulex* complex has been intensively studied due to its dominance in freshwater habitats in North America, and its variation in reproductive mode and ploidy level. *Daphnia* usually reproduce by cyclic parthenogenesis, which is clonal reproduction interrupted by bouts of sexual reproduction. However, some lineages reproduce by obligate parthenogenesis [14-18]. The *D. pulex* complex includes seven species that have been distinguished on the basis of morphological, ecological, and genetic data [19-22]. In North America, two species of the complex, *D. pulex* and *Daphnia pulicaria*, are dominant in freshwater habitats, and are morphologically similar but ecologically distinct [19], although they hybridize in nature [18,23-25]. Diploid hybrids always have *D. pulex* mitochondrial genomes (mtDNA) and have been found to reproduce by obligate parthenogenesis in nature based on patterns of allozyme variation [18,26] and laboratory crosses [26], but see [27] for experimentally produced hybrids capable of sexual reproduction. Analyses of allozyme variation indicate that introgression is rare or nonexistent in areas where the two species co-occur [16,18,25] but a recent study using mitochondrial and nuclear markers has shown that introgression between these species had a substantial impact on their evolutionary history [28].

Hybridization has also occurred among other species in the *D. pulex* complex [29-32]. For example, hybridization between North American species and circumarctic or European *Daphnia* species has been suggested based on allozyme, mtDNA or nuclear markers such as the ribosomal intergenic spacer (IGS) [21,33,34]. Some hybrids found in arctic and subarctic regions are polyploids (mainly triploids [35]). Polyploids with *D. pulex*, *D. pulicaria* and *Daphnia middendorffiana* (a circumarctic species) mtDNA are thought to have arisen from hybridization between *D. pulex* and *D. pulicaria*, or with another species which no longer exists as a cyclic parthenogen [22,29,30,32]. Another group of polyploids has been found in *Daphnia tenebrosa*, a circumarctic species [31] and includes both triploid and tetraploid clones [35] but their hybrid nature is unknown [22].

The *D. pulex* genome has recently been sequenced [36] and numerous Class II DNA transposons have been identified from it [37]. One Class II element, *Pokey* (Subclass I; *sensu*[38]) of the *piggyBac* superfamily, has been extensively studied in non-hybrid populations. It inserts in a site-specific fashion into a unique TTAA site in the tandemly repeated 28S rRNA genes [39-41] and is also encountered in other parts of the genome. Two *Pokey* elements from *D. pulicaria* have been completely sequenced [39] and show that the element has 16 bp imperfect terminal inverted repeats (TIRs) and encodes a putative transposase that is much longer than originally estimated and contains an intron (Y. Bigot, personal communication, [40]). In addition, the non-coding region upstream of the transposase gene contains a repeated sequence that is similar to a sequence in the unique region of the host’s ribosomal IGS [34,39,40]. Based on patterns of polymorphism observed among natural populations, previous studies [41,42] have suggested that *Pokey* may be active in sexual populations of *D. pulex* but not in obligate parthenogens. Although Class II DNA transposons are more prone to transfer horizontally between lineages than retrotransposons, horizontal transfer does not seem to play a major role in the evolution of *Pokey* as 28S rRNA genes and mtDNA phylogenies of species in the subgenus *Daphnia* are congruent with the phylogeny of *Pokey* elements [7] leading to the conclusion that *Pokey* elements may co-evolve with their hosts.

The purpose of the present study is to describe *Pokey* sequences in hybrid isolates from the *D. pulex* complex. Specifically, we aim to determine if *Pokey* sequences inserted in rRNA genes reflect the putative parental species origins of the hybrid isolates. As *Pokey* elements insert in 28S rRNA genes, they are subject to genetic processes such as inter-allelic recombination, gene conversion and unequal crossing over [43] that may lead to the concerted evolution of these genes. If *Pokey* elements are strictly vertically inherited and if recombination is negligible, we expect that a phylogeny of *Pokey* elements should follow the host phylogeny, and *Pokey* elements encountered in hybrid genomes should correspond to those encountered in their host parental species. If recombination is important in *Pokey* evolution and has occurred between elements showing recent activity and after hybridization, hybrid isolates will carry either sequences inherited from both parents or mosaic sequences between parental types. Alternatively, if recombination occurs between inactivated elements that diverged in the distant past, we could see *Pokey* mosaic sequences that do not necessarily reflect the progenitor species of the hybrid isolates in the *D. pulex* complex.

After cloning and sequencing partial *Pokey* elements from representatives of species in the *D. pulex* complex, we: (a) detected recombinant elements and located the recombination breakpoints in each, (b) estimated recombination rate parameters across sequences using a coalescent based method, (c) determined parental sequences from which the recombinant elements arose and (d) reconstructed the evolutionary history of the *Pokey* sequences. Moreover we used a PCR-RFLP protocol to search for the presence of different *Pokey* elements in additional hybrid and non-hybrid isolates of the *D. pulex* complex across freshwater habitats and geographical locations.

## Methods

### *Daphnia* samples

We analyzed 51 isolates representing six of the seven lineages of the *D. pulex* complex identified by Colbourne *et al*. [20]. Only *D. melanica* was not included. Nine *Pokey* sequences were obtained from nine isolates studied by Penton and Crease [7]. We established parthenogenetic lines (isolates) in the laboratory from 42 individual females sampled from nature between 2004 and 2010 (see Additional file 1) and cultured them using standard techniques [44]. For 50 of these isolates, genomic DNA was extracted from 10 to 30 individuals weighing approximately 100 mg (wet weight) using the DNeasy Tissue kit (QIAGEN Inc., Mississauga, ON, Canada) according to the supplier’s protocol. Species identity of these isolates was assessed by combining information on morphology, haplotype of the mitochondrial ND5 gene and genotype of the nuclear *Lactate dehydrogenase* (*Ldh*) gene as described in [22]. Ploidy levels were previously assessed using microsatellite genotypes and flow cytometry [35].

### Structure of the *Pokey* transposase gene

RT-PCR was performed on RNA samples extracted from a sexual *D. pulex* isolate to determine if *Pokey* transposase transcripts were present and to provide further evidence for the presence of an intron in the transposase gene. RNA was extracted from the parthenogenetic offspring of one sexual *D. pulex* isolate PX2-ON-9 (provided by Dr. M. Cristescu at the University of Windsor). The tissue was stored in RNA Later (Qiagen) at −20°C and RNA was extracted using the RNAqueous-4PCR kit (Life Technologies Inc., Grand Island, NY, USA) and standard manufacturer’s protocols. The absence of DNA contamination was verified using standard PCR and *Pokey* transposase primers. Reverse transcription of the RNA used the SuperScript III One-Step RT-PCR with Platinum *Taq* Kit (Life Technologies) and standard manufacturer’s protocols. Primers used were Pok4065F (5’-TGATTCACCGAGGCCTCAGTTC) with Pok4488R (5’-GAATCGCTCGCGAGTCATGG) and Pok5026F (5^′^-TCGAACCTGCAGCCGGACGAATTTGCAG) with Pok5985R (5’-CACGTCGGTTAGAATATTCTGGCTCGTCGG). Numbers correspond to nucleotides in the 6.6 kb element from *D. pulicaria* (GenBank accession # AY115589.1, [8]).

### Cloning and sequencing *Pokey* alleles

To examine the impact of hybridization on *Pokey* diversity, *Pokey* alleles inserted in 28S rRNA genes from various isolates of the *D. pulex* complex were cloned and sequenced. An approximately 1600 bp fragment of *Pokey* from 16 isolates (2 diploid hybrids, 5 polyploid hybrids, 1 introgressed polyploid (TE3-MB-4), 3 diploids, 3 polyploids with unknown hybrid state, 3 diploid non-hybrids) of the *D. pulex* complex (see Additional file 1) were amplified using Pok5026F and the primer 28SR (5^′^-TCCATTCGTGCGCGTCACTAATTAGATGAC), which is located 46 bp downstream of the *Pokey* TTAA target site. PCR reactions were performed using the Phusion™ high-fidelity PCR kit (Finnzymes, Woburn, MA, USA) according to the supplier’s protocol. The thermocycling profile consisted of 1 cycle of 1 minute at 94°C, 35 cycles of 30 seconds at 94°C, 30 seconds at 55°C and 2 minutes at 72°C, with a final incubation of 5 minutes at 72°C. PCR amplicons were cloned using the StrataClone™ Blunt PCR Cloning Kit (Agilent Technologies Inc., Santa Clara, CA, USA) according to the supplier’s protocol. For each isolate, 10 to 12 plasmids chosen randomly from a minimum of 40 isolated colonies were purified using the E.Z.N.A. Plasmid Mini Kit II (Omega Bio-tek Inc., Norcross, GA, USA). *Pokey* sequences obtained from these plasmids were named using the labels of the isolate from which they were cloned plus the number that corresponds to the colony from which they were picked (for example PX2-QC-8_28 is the *Pokey* allele isolated from colony 28 produced during the cloning of PX2-QC-8 *Pokey* sequences). These *Pokey* alleles were compared to *Pokey* sequences from a previous study which focused on non-hybrid isolates [7] to ensure that the elements analyzed in this study belong to the *Pokey* lineage. Partial *Pokey* sequences were manually aligned using the ClustalW module of BioEdit v.7.0.5 [45]; available at http://www.mbio.ncsu.edu/BioEdit/bioedit.html.

### Detecting putative recombination events

Potential recombination events among *Pokey* alleles were explored in a dataset of 53 sequences in which ambiguous sites and indels were deleted to prevent identification of false recombination events. The phi-test [46], implemented in Splitstree v4.10 [47], and GARD (Genetic Algorithm for Recombination Detection, available via the DataMonkey website at http://www.datamonkey.org[48]) were used to determine if recombination events may be detected in our *Pokey* sequence dataset as well as in partial transposase sequences included in them.

Hudson and Kaplan minimum recombination events, R_m_[49] were calculated with the DnaSP v5 software [50]. Moreover we used kwarg (available at http://www.stats.ox.ac.uk/~lyngsoe/section26/), a heuristic alternative to the branch and bound method implemented in beagle [51], to calculate R_h_[52], which gives an estimate of the lower bound of the minimum number of recombination events. Kwarg does not guarantee that the minimal recombination history will be found but returns a history with a low number of recombination events under the infinite sites assumption. We ran kwarg 2,048 times with the default scoring scheme and recorded the number of recombination events for each iteration.

### Recombination rate parameter

As all the recombination analyses detected recombi nation events (see Results), we estimated *Pokey*‘s population recombination rate parameters along the sequence using a full likelihood coalescent approach based on a finite-sites model implemented in LAMARC v2.1.6 [53]. This approach estimates the parameter *r*_*LAM*_*= c/μ* where *c* is the recombination rate per site per generation and *μ* is the neutral mutation rate per site per generation. This model considers that (a) all recombination is homologous, (b) there is no association between recombination frequency and sequence divergence, (c) gene convergence and interference did not occur, (d) recombination events are selectively neutral and (e) recombination rate does not vary along the sequence. The F84 evolutionary model was used as it is a closer fit to the substitution model, Tamura 3-parameter with gamma shape and invariant sites, inferred with MEGA5 [54] than the other model (GTR) offered by LAMARC. The F84 model is also computationally faster. To account for substitution rate differences between non-synonymous sites and all other sites, two categories of relative mutation rate were assigned. Our sampling strategy included 20 initial chains of 20,000 genealogies and two final chains of 1,000,000 genealogies with 2,500 samples discarded per chain. Adaptive heating was used to improve the search of parameter space with initial relative temperature of 1, 1.1 and 2. The entire analysis was replicated four times and the results were combined using the algorithm of Geyer [55]. Coalescent-based analyses in LAMARC estimate both genetic diversity at the population level, *θ*_*LAM*_ = *4N*_*e*_*μ* (where *μ* is the mutation rate per site for a diploid population and *N*_e_ is the effective population size) and the recombination rate *r*_*LAM*_ = *c*/*μ* (where *c* is the rate of recombination per site per generation). Therefore, the population recombination rate parameter can be calculated as *R* = *θ*_*LAM*_*r*_*LAM*_ = (4*N*_*e*_*μ*)(c/*μ*) = 4*N*_e_*c*.

### Detection of recombination breakpoints

Nucleotide diversity among sequences (that is, π and θ) and mutation rate heterogeneity along sequences (the Gamma shape parameter, α) may have a dramatic impact on the performance of different methods to detect recombination breakpoints [56-58]. For example, Posada and Crandall [57] have shown that different methods may have a different propensity to detect recombination when nucleotide diversity varies and mutation rate heterogeneity is fixed. Moreover, they have shown that mutation rate heterogeneity may lead some methods to detect false positives [56-58].

We estimated nucleotide diversity (π and θ) using DnaSP v5.10.01 [50]. Mutation rate heterogeneity (α) and a nucleotide substitution model were inferred using a maximum likelihood framework in MEGA5 [54]. To detect recombination breakpoints and identify which parental sequences formed the recombinant alleles, we applied a stepwise protocol of the maximum chi-square method [59,60] using the stepwise package v0.1.1 (available at http://stat.sfu.ca/statgen/research/stepwise.html). The maximum chi-square method is a sliding window approach that computes a chi-square statistic from a 2 x 2 table with counts of matches and mismatches between the two half windows separated by a proposed breakpoint. As high mutation rate heterogeneity in *Pokey* sequences (see Results) may lead to false negatives when window half-widths are too small, we used different values of window half-width (70, 80, 90 and 100 nucleotides) and 100,000 Monte Carlo replicates for the permutation distribution. The maximum chi-square analysis was reiterated including breakpoints detected by previous steps, as the stepwise protocol specifies, until no further breakpoint was detected. This maximum chi-square analysis identifies two sequences involved (one derived, one parental) for each recombination event detected. Recombination breakpoint detection by the maximum chi-square method is relatively more powerful and less prone to type I error (false positives) than other algorithms [56,57] when sequences are subject to high mutation rate heterogeneity. Each event detected between different sequence pairs was checked by eye.

### Phylogenetic analyses and ancestral recombination graphs

Dendrograms were produced for each fragment separated by recombination breakpoints highlighted by GARD analysis of the 53 sequences using the Neighbor-joining algorithm [61]. We constructed a phylogenetic network using the NeighborNet algorithm implemented in Splitstree v4.10 [47]. To understand more accurately the process and evolutionary history of recombination in the *Pokey* fragments cloned here, we constructed an ancestral recombination graph using kwarg [51,52,62]. We chose the simulation with the lowest R_h_ value to build this graph.

### RFLP analysis

Given the intra- and intergenomic variability of *Pokey* alleles in isolates from which elements were cloned, and the high number of recombination events in these sequences (see Results), we aimed to explore the presence/absence of these alleles in additional isolates belonging to the *D. pulex* complex. To do this, we developed a restriction enzyme analysis (that is, RFLP) based on *Pokey* sequences analyzed so far and the recombination breakpoints detected by the GARD analysis. We then screened *Pokey* alleles from 41 isolates (see Results) for RFLP haplotypes. PCR reactions (two replicates for each isolate) were performed in 25 μL reaction mixtures containing 1x Econotaq PCR buffer, 2 mM MgCl_2_, 50 μM dNTP, 0.1 μM of each primer (Pok5026F and 28SR), and 1 unit of EconoTaq polymerase (Lucigen Corporation, Middleton, WI, USA) or using the Phusion™high-fidelity PCR kit according to the manufacturer’s protocol (Finnzymes). PCR amplifications were performed using the thermocycling profile described above. Restriction enzymes that cut at specific sites in some *Pokey* alleles may be used to highlight *Pokey* sequences that have undergone recombination events. We used NebCutter v2.0 (http://tools.neb.com/NEBcutter2/) on each of the 53 sequences to choose enzymes that would highlight different pure and recombinant *Pokey* alleles. The restriction enzymes *Dra*I, *Bsp*HI and *Bst*EII (New England BioLabs Inc., Ipswich, MA, USA) were used in a two-step protocol. *Dra*I, *Bsp*HI and *Bst*EII enzymes cut at 380 bp, approximately 790 bp, and approximately 1080 bp. The eight possible conformations were differentiated on a 3% agarose gel. In the first step, *Dra*I and *Bsp*HI were used in the same mix containing NEBbuffer4 and digestion was conducted at 37°C for two hours. In the second step, NEBbuffer3 at a 1X final concentration, BSA at 1U/μl and *Bst*EII at 1U/μl were added to the solution and digestion was conducted at 60°C for one hour. Digested PCR products were separated on 3% agarose gels for two hours at 95 volts.

Cloning and PCR amplifications may produce chimeric sequences (for example, recombinants between alleles) and lead to an overestimation of recombination in nature. We assessed this hypothesis by re-amplifying *Pokey* sequences from isolates used in our phylogenetic survey using the Phusion^TM^ high-fidelity PCR kit and by increasing elongation steps of the thermocycling profile to three minutes instead of two minutes. Increasing the elongation steps should reduce the probability of producing chimeric sequences [63]. Results were compared to PCR-RFLP results with two-minute elongation steps to determine if the recombination pattern is reduced. Moreover, if recombination events identified in the data are not artifacts, PCR-RFLP results should be concordant with expectations based on the cloned elements that we sequenced.

## Results

### Structure of the *Pokey* transposase gene

**Figure 1:**
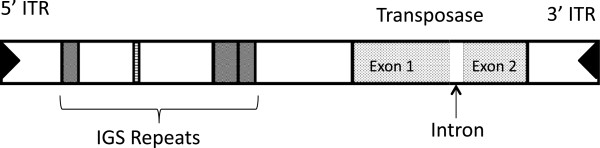
**Schematic view of a *Daphnia Pokey *element.** IGS repeats (gray boxes) are sequences similar to a region of the *D. pulex* intergenic spacer. 5^′^- and 3^′^-ITR are imperfect 16 bp inverted terminal repeats. IGS, intergenic spacer.

Sequencing of RT-PCR products, performed on RNA extracted from a sexual isolate of *D. pulex* (PX2-ON-9, Additional file 1), showed that a sequence upstream of the proposed stop codon in the 6.6 kb *Pokey* element is actually the beginning of a 68 bp canonical GT-AG intron. When spliced out, this adds another exon (exon 2) and extends the transposase coding region by 585 bp (Figure 1).

### Recombination signals

Forty-four sequences of the 3’ end of *Pokey* elements were added to nine *Pokey* alleles previously sequenced from non-hybrid isolates for a total of 53 sequences analyzed. These sequences have variable lengths due to multiple indels and vary from 1,419 bp (PX2-QC-8_30) to 1,471 bp (TE3-MB-4_9). These sequences contain two exons of the *Pokey* transposase gene separated by an intron of 68 bp to 70 bp. Only the last 259 bp of exon 1 is included, but exon 2 is complete and varies in length from 585 bp to 627 bp. Nucleotide diversity estimates based on the full-length *Pokey* sequences are π = 0.048 and θ = 0.037, and the gamma shape parameter (*α*) is 0.8423 with 63.3% of sites being invariant. Nucleotide diversity in exon 2 is an order of magnitude higher than exon 1 and about twice as high as diversity in the non-coding region downstream of the transposase gene (Table 1). Total amino acid (aa) sequences encoded by the two exons range from 282 aa in most sequences to 297 aa with most of the length variation (196 aa to 210 aa) occurring in exon 2. One sequence (TE3-MB-1_14) has a one base pair insertion that causes a frameshift mutation in the first exon.

**Table 1:** **Nucleotide diversity and mean similarity between sequence pairs for each part of the *Pokey *sequences**

	**Coding**			**Non-coding**		
	**Total**	**exon 1**	**exon 2**	**Total**	**intron**	**3**^**′**^**NCR**
π	0.055	0.007	0.076	0.038	0.038	0.038
θ	0.037	0.009	0.049	0.036	0.033	0.036
Mean (%)	94.2	99.3	93.1	96.0	96.0	96.0

3^′^NCR, non-coding region downstream of the transposase gene.

**Figure 2:**
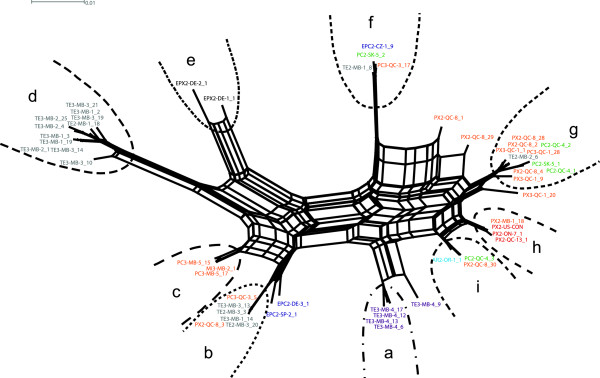
**Phylogenetic network of 53 partial *Pokey *sequences from isolates of the *Daphnia pulex *complex.** The network was constructed using the NeighborNet algorithm. Dashed lines delineate sequence groups presented in Table 2. Colors represent species or hybrid state of isolates: red = *D. pulex*, light blue = *D. arenata*, grey = *D. tenebrosa*, black = European *D. pulex*, dark blue = European *D. pulicaria*, green = *D. pulicaria*, orange = putative pulex-pulicaria hybrids, purple = the isolate with *D. tenebrosa* mtDNA and a *D. pulex* nuclear genome.

Recombination analyses performed on the 53 *Pokey* sequences, as well as in the reduced dataset (about 852 bp to 891 bp in length) of 53 partial transposase sequences, showed evidence of recombination events (phi-test; *P* <0.01). Two recombination breakpoints were found using GARD analysis of the entire sequences (at bp 540 and bp 820; Kishino-Hasegawa-tests, *P* <0.001 for each breakpoint) and in the transposase coding region dataset (at bp 498 and bp 686; Kishino-Hasegawa-tests, *P* <0.001 for each breakpoint). Six of the seven recombination events detected by the chi-square method are encountered in exon 2 (see Additional file 2). One step, using the stepwise protocol of the chi-square maximum method with the different half width windows (70, 80, 90 and 100), was sufficient to detect at least 20 recombination events, named A to T (see Additional file 3). The intricate way in which the numerous recombination events shape the evolution of *Pokey* elements led us to identify groups of sequences that we named **a**, **b**, **c**, **d**, **e**, **f**, **g**, **h** and **i** (see Additional file 4). The phylogenetic network generated from 53 partial *Pokey* sequences shows clear groups of sequences (Figure 2 and Additional file 4). Six sequences (TE3-MB-4_9, PX2-QC-8_1, PX2-QC-8_29, PX3-QC-1_20, EPC2-SP-2_1 and EPC-DE-3_1) were not included in groups as they may have originated from different recombination events than did the sequences with which they cluster in the network (Table 2 and Figure 2). Sequences amplified from some isolates belonging to the same species clustered together, each in a distinct group (for example, *D. tenebrosa* group **d**, European *D. pulex* group **e**, *D. pulex* group **h***,* Figure 2). Sequences from the isolate TE3-MB-4 (**a** + TE3-MB-4_9) and sequences from triploid isolates from Churchill (PC3-MB-6 and MI3-MB-2, group **c**), which have *D. pulicaria* or *D. middendorffiana sensu stricto* mtDNA haplotypes [35], also form separate groups. Various sequences amplified from *D. pulicaria* (PC2-QC-4 and PC2-SK-5) or hybrid isolates (PX2-QC-8, PX3-QC-1 and PC3-QC-1) clustered together in group **g**. Three groups included sequences amplified from isolates that belong to different species (Figure 2). Two groups (‘**b** + EPC2-SP-2_1 + EPC-DE-3_1’ and **f**) included sequences from pulex-pulicaria hybrid isolates (PX2-QC-8 and PC3-QC-3), *D. tenebrosa* isolates (TE3-MB and TE2-MB) and European *D. pulicaria* isolates (EPC2-SP-2 and EPC-DE-3). Group **i** is represented by sequences from *D. arenata* (AR2-OR-1), *D. pulicaria* (PC2-QC-4) and a pulex-pulicaria hybrid (PX2-QC-8). An allele from one *D. tenebrosa* isolate (TE2-MB-2) grouped with sequences from pulex-pulicaria hybrid isolates in group **g**.

**Table 2:** **Recombination events in which groups of sequences (see text for more explanation) are involved according to maximum *χ***^**2 **^**and GARD dendrogram analyses**

**Sequences**	**Events involving sequences according to the maximum *χ***^**2 **^**test (other sequences involved**^**a**^**)**	**Pairs of probable parental sequences estimated using GARD dendrograms based on two different recombination breakpoints**^**b**^
**a**	**I** (**h**, **g**, **c**, PX2-QC-8_29), **IV** (**b**, EPC2-SP-2_1), **V** (**h**), **VII** (**g**)	**h : c** or **i** : **c**
TE3-MB-4_9	**I** (**i**), **VII** (**g**, PX2-SF-8_29, PX2-SF-8_1, **f**)	**h : c** or **i** : **c** and **c** : **h**
**b**	**II** (**i**), **IV** (**a**)	**d** : **a** or EPC2-SP-2_1 **: a** or EPC2-DE-3_1 **: a**
		**d** : **c** or EPC2-SP-2_1 **: c** or EPC2-DE-3_1 **: c**
		**d** : **i** or EPC2-SP-2_1 **: i** or EPC2-DE-3_1 **: i**
EPC2-SP-2_1	**II** (**i**), **IV** (**a**, **h**)	**b : i**, **b : c** or **b : a**
EPC2-DE-3_1	-	**b : i**, **b : c** or **b : a**
**c**	**I** (**a**), **IV** (**i**), **V** (**h**), **VI** (**g**)	**d : i**
**d**	-	-
**e**	-	-
**f**	**VII** (TE3-MB-4_9)	-
PX2-QC-8_29	**I** (**a**), **VII** (TE3-MB-4_9)	**i : f** or **h : f**
PX2-QC-8_1	**VII** (TE3-MB-4_9)	**g** : **f**
**g**	**I** (**a**), **VI** (**c**), **VII** (**a**, TE3-MB-4_9)	**unknown** : **f**
PX3-QC-1_20	**III** (**i**)	**g : h** or **g : i** and **i : g** or **h : g**
**h**	**IV** (EPC2-SP-2_1), **V** (**a**, **c**)	**i : Unknown** or **a : Unknown**
**i**	**I** (TE3-MB-4_9), **II** (b, EPC2-SP-2_1), **III** (PX3-QC-1_20), **IV** (c)	**h : Unknown**

^a^Multiple sequences between parentheses highlight probable parental sequences for the same recombination event; ^b^The first sequence in each pair corresponds to the sequence before the estimated recombination breakpoint. GARD, Genetic Algorithm for Recombination Detection.

Groups of sequences with the same recombination site may represent a single recombination event between ancestral sequences. For example, the recombination breakpoint of events A (**a** : **c)**, B **(a** : **h)**, C **(a** : PX2-QC-8_29), D (**j** : TE3-MB-4_9) and E (**i** : **a)** were estimated to be bp 592 to bp 607 for all pairs. Thus, we considered them to be the same recombination event, **I**. This approach results in seven different recombination events named **I** (bp 592 to bp 607), **II** (bp 748 to bp 800), **III** (bp 815 to bp 856), **IV** (bp 825 to bp 856), **V** (bp 917 to bp 920), **VI** (bp 952 to bp 957) and **VII** (bp 1,038 to bp 1,073) (see Additional file 4). Although it is still possible, it seems unlikely that all recombination events between sequence pairs were independent and not due to past events in the common ancestors of groups of sequences.

The Hudson-Kaplan minimum number of recombination events (R_m_) in our partial *Pokey* sequences dataset is 30 according to the algorithm implemented in DnaSP v5.10.01. According to the kwarg algorithm, R_h_, the estimated lower bound of the minimum number of recombination events, follows a normal distribution with mean 96.39 and a standard deviation of 3.10.

### Recombination rate

Using LAMARC to co-estimate mutation rate *θ*_*LAM*_ = 5.94 × 10^-2^ (with 95% support intervals of 3.63 × 10^-2^ and 8.16 × 10^-2^) and the overall recombination rate *r*_*LAM*_ = 5.09 × 10^-1^ (with 95% support intervals of 3.57 × 10^-1^ and 7.68 × 10^-1^) allowed us to estimate a population recombination rate of 3.02 × 10^-2^ recombination events per site per generation for *Pokey* elements.

### Ancestry of recombinant *Pokey* sequences

**Figure 3:**
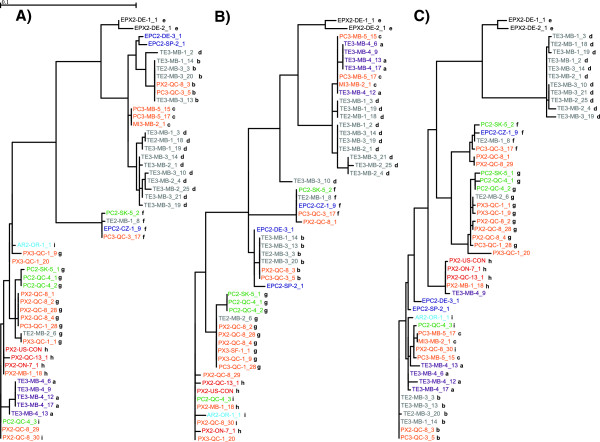
**Unrooted dendrograms of 53 partial *Pokey *sequences from isolates of the *Daphnia pulex *complex.** (**A**), (**B**) and (**C**) represent trees constructed from three regions of recombinant *Pokey* sequences (bp 1 to approximately bp 540, bp approximately 540 to approximately bp 820, and bp approximately 820 to approximately bp 1,450, respectively) defined by recombination breakpoints identified by GARD analysis. Colors represent species or hybrid state of isolates: red = *D. pulex*, light blue = *D. arenata*, grey = *D. tenebrosa*, black = European *D. pulex*, dark blue = European *D. pulicaria*, green = *D. pulicaria*, orange = putative *pulex-pulicaria* hybrids, purple = the isolate with *D. tenebrosa* mtDNA and a *D. pulex* nuclear genome. GARD, Genetic Algorithm for Recombination Detection.

Recombination breakpoints estimated by the GARD analysis (at bp 540 and bp 820) seem to correspond to the recombination breakpoints of events **I** and **IV** estimated using the maximum chi-square analysis (at bp 592 to bp 607 and bp 825 to bp 856, respectively). Dendrograms (Figure 3) were produced from each fragment (from bp 1 to bp 540, from bp 541 to bp 820, and from bp 821 to bp 1,450, respectively) of the *Pokey* sequences bound by recombination breakpoints identified by the GARD analysis. Combining the dendrogram analysis (Figure 3) and the maximum chi-square method of recombination breakpoint detection (see Additional file 3) allowed us to estimate from which parental sequences the recombinant *Pokey* sequences originated. Recombination events and probable parental sequences of each group of sequences are summarized in Table 2. While groups **d**, **e** and **f** do not seem to derive from any recombination event (Figure 3, Table 2), other sequences may have originated from multiple events (TE3-MB-4_9 and PX3-QC-1_20; Table 2). Moreover, some sequences may have originated from recombination events between sequences that were themselves recombinant. For example, sequences from group **a** originated from recombination between sequences from groups **h** or **i** and from group **c**. This last group may originate from a recombination event between sequences from groups **d** and **i** (Table 2, Figure 3).

Non-synonymous polymorphism at the nucleotide level leads to a high level of polymorphism at the aa level and, coupled with recombination events, produces a diversity of partial aa sequences of the *Pokey* transposase (see Additional file 2).

### Ancestral recombination graphs

Additional file 5 shows an ancestral recombination graph with 89 recombination events (R_h_) identified by the Song and Hein algorithm [52]. The evolutionary history of *Pokey* allele TE3-MB-4_12, which contains 38 recombination events, is highlighted in red. This ancestral recombination graph shows putative past recombination events that were not detected by the maximum chi-square method. For example, the evolution of EPX2-DE-1_1, encountered in a European *D. pulex* isolate, seems to involve 25 past recombination events.

### *Pokey* alleles in the v1.1 *Daphnia* genome

The v1.1 genome sequence of *D. pulex*[36] was scanned for our partial *Pokey* sequence using TBLASTN to explore which alleles are present. Sequences from contigs with E-value = 0.0 and without indels more than 10 nucleotides long were aligned with our 53 sequences to generate a dataset of 58 sequences. A NeighborNet phylogenetic network (see Additional file 6) shows that *Pokey* elements from the genome sequence group with sequences encountered in diploid and polyploid isolates from the pulicaria group (PC2-SK-5 and PC3-QC-3), in diploid *D. tenebrosa* (TE2-MB-1) and in European *D. pulicaria* (EPC2-CZ-1).

### RFLP analysis of *Pokey* alleles in additional isolates

To test if recombinant *Pokey* alleles are encountered in additional clones of the *D. pulex* complex, we used a restriction enzyme analysis (that is, PCR-RFLP) of alleles amplified from the 28S rRNA genes from 41 isolates (see Additional file 7). Eight different haplotypes were found (numbered 1 to 8) using three restriction enzymes, some of which correspond to sequences from the groups encountered in our phylogenetic analysis. The distribution of these haplotypes is summarized in Table 3. We found that rRNA genes from different genomes may contain up to five different *Pokey* RFLP haplotypes with an average of 1.74 per isolate and a standard deviation of 0.96.

**Table 3:** Taxonomic, habitat and geographical distributions of RFLP haplotypes

**Haplotype**	**Percentage and taxonomic occurrence**	**Habitat**	**Geographic location**
1	33.33% of isolates, mainly with *D. pulex* mitochondria	ponds	QC, ON, MB (CAN), MI (USA)
2	38.09% of isolates, *D. pulicaria*, polyploids with *D. pulicaria* mitochondria, diploid/triploids with *D. pulex* mitochondria and one *D. tenebrosa* isolate	Various habitats	various locations
3	26.19% of isolates, *D. pulex,* diploid hybrids with *D. pulex* mitochondria and triploid hybrids *D. pulicaria* mitochondria, European *D*. pulicaria and *D. tenebrosa* isolâtes	various habitats	various locations
4	30.95% of isolates, *D. pulex,* diploid hybrids with *D. pulex* mitochondria and triploid hybrids *D. pulicaria* mitochondria, *D. middendorffiana*, European *D*. pulicaria and *D. tenebrosa* isolates	various habitats	various locations
5	One introgressed isolate (TE3-MB-4)	rock bluff pools	Churchill MB, (CAN)
6	9.76% of isolates, *D. pulex*, *D. pulicaria* or *D. middendorffiana* and *D. pulex*-*D. pulicaria* hybrids	ponds or rock bluff pools	Kujjuarappik and Metis, QC (CAN) Churchill, MB (CAN)
7	One *D. pulex*-*D. pulicaria* hybrid (PX2-MI-11)	ponds	MI (USA)
8	26.8% of isolates, *D. pulex* and *D. pulicaria*	various habitats	various locations

## Discussion

### Hybridization among *Daphnia* species

Our results show that recombination has a dramatic impact on the evolution of *Pokey* alleles in the rDNA of *Daphnia*. The recombinant sequences do not seem to be due to cloning artifacts as some isolates may have just one recombinant *Pokey* allele in their genome (for example, triploid isolates PC3-SK-5 and PC3-MB-4 or diploid isolates EPC2-CZ-1, EPC2-SP-2 and PX2-QC-1, Additional file 7). Increasing the extension time of the PCR cycles to avoid artefactual recombination events during amplification did not change the RFLP results (data not shown). Moreover, *Daphnia* isolates show RFLP patterns that are concordant with those expected from the cloned *Pokey* sequences (see Additional file 7).

*Pokey* sequences show substantial nucleotide diversity (π = 0.048 and θ = 0.037, Table 1), undergo recombination at a rate of 3.02 × 10^-2^ events per site per generation and rarely spread by horizontal transfer [7]. As predicted in the case of recombination events between *Pokey* elements that co-evolve with their host and have undergone recent activity, hybrid isolates from the *D. pulex* complex carry either sequences inherited from putative parental species or mosaic sequences produced by recombination between parental types. Most *Pokey* sequences from pulex-pulicaria hybrids (for example, PX2-MB-1, PX3-QC-1_1, PC3-QC-1_28 or PX2-QC-8_28) either cluster with *Pokey* alleles from *D. pulex* (group **h**) or *D. pulicaria* (PC2-QC-4 and PC-SK-5_2 in group **g**) in the phylogenetic network (Figure 2) or are recombinants between sequences from these groups (that is, PX3-QC-1_20). *Pokey* sequences from polyploid isolates with mtDNA from either *D. pulicaria* or *D. middendorffiana* from Manitoba, Canada seem to be recombinants between sequences found in *D. tenebrosa* (group **d**) and *D. pulicaria*, *D. pulex* or *D. arenata* (that is, groups **g**, **h** or **i,** respectively, Table 2). This suggests that *D. tenebrosa* may be one of the parental species of these polyploid isolates, which are thought to originate from hybridization between *D. pulex* and *D. pulicaria* or with another species that no longer exists as a cyclic parthenogen [22,29,30,32]. Phylogenetic analysis of *Ldh*A sequences is also concordant with a hybrid origin of polyploid isolates with *D. middendorffiana* mtDNA (Figure 3 in [64]).

The introgressed triploid isolate, TE3-MB-4, which has *D. tenebrosa* mtDNA and most likely a *D. pulex* nuclear genome [22,31]), carries alleles from group ‘**a** + TE3-MB-4_9’ with a minor trace from *D. tenebrosa* and major traces from *D. pulex* or *D. pulicaria* alleles. The presence of alleles with traces of *D. pulex* or *D. pulicaria* in *D. tenebrosa* or European *D. pulicaria* isolates (TE3-MB-1, TE3-MB-3, TE2-MB-1, TE2-MB-3, EPC2-MB-2 and EPC2-MB-3) may be a remnant of hybridization events between these four species. Similarly, *Pokey* alleles from European *D. pulicaria* isolates (EPC2-SP-2_1 and EPC2-DE-3_1) also seem to be recombinants between alleles from North American *D. pulex* or *D. pulicaria* and *D. tenebrosa* (recombination events G, K and L in Table 2 and Figure 2). Although Vergilino *et al*. [22] did not detect evidence of hybridization between species in the pulicaria group (North American *D. pulex* and *D. pulicaria*) and the tenebrosa group (*D. tenebrosa* and European *D. pulicaria*) using microsatellite loci, Weider *et al*. [33] did find evidence for it using allozyme data. Moreover, Ambrose and Crease [34] have shown that IGS segments from some isolates of European *D. pulex* may have originated from North American *D. pulicaria*. As North American *D. pulex* and *D. pulicaria* have been found in Europe [21], introgression of *Pokey* alleles from North American species into European species is possible and may generate recombinants that persist for long periods of time and are able to spread over broad geographic areas.

Although most *Pokey* sequences from hybrid isolates are mosaics, which is consistent with recent recombination between alleles from different parent species, some recombinant alleles may be more ancient and represent retained ancestral polymorphism (that is, incomplete lineage sorting). The clustering of recombinant alleles in group **b**, which are encountered in *D. tenebrosa* isolates, a hybrid *D. pulex* (PX2-QC-8) and a polyploid with *D. pulicaria* mtDNA (PC3-QC-3) suggest that these recombinant alleles are ancient.

*Pokey* alleles from the isolate whose genome was sequenced [36] cluster with alleles from group **f** (Figure 3 and Additional file 6) whereas *Pokey* alleles from other *D. pulex* isolates cluster in group **h**. This may be the result of incomplete lineage sorting or a signature of past hybridization events that have occurred in the population from which the genome isolate was sampled. Alternatively, the genomic *Pokey* alleles may have a different evolutionary history than their paralogs in rDNA. Moreover, these genomic alleles show a limited signature of recombination according to the GARD analysis. As the recombination rate is variable across the *Daphnia* genome [65,66], we may expect this result if *Pokey* elements are inserted in genomic regions showing low recombination rates.

### Recombination parameters among *Pokey* elements

The number of recombination breakpoints detected in our sampled sequences by different methods was highly variable. Most recombination breakpoint detection algorithms underestimate the number of recombination events [57] and their accuracy varies depending on whether the method uses summary statistics or the maximal information in the sample [58]. The maximum chi-square method performed in a stepwise manner and for different window sizes detected a minimum of seven unique recombination events between *Pokey* alleles from different species of the *D. pulex* complex (see Additional file 3, Table 2). This value is likely to be an underestimate as a high recombination rate may lead to overlapping recombination events. As the maximum chi-square is a substitution-based method that compares substitution distributions from two windows flanking a tested site, recombination breakpoint detection will be dependent on the size of the windows used when mutation rate heterogeneity is high and/or nucleotide diversity is low. Small windows should increase the probability of detecting recombination breakpoints in high diversity regions but may be unable to detect recombination events in low nucleotide diversity regions. Increasing the size of the windows to increase recombination detection accuracy in regions with low nucleotide diversity may lead to less accuracy in the regions with multiple recombination events. The estimate of Hudson-Kaplan minimum recombination events (R_m_) is 30 and the haplotype lower bound (R_h_) estimate is 96.39 ± 3.10. R_m_ is a parsimony-based method (that is, the four gamete test) and may underestimate the actual lower bound of recombination events, particularly when the recombination rate is high [67,68]. In contrast to the former estimators, R_h_, estimated using a maximum-likelihood framework that uses the maximal information in the sample, is probably more reliable than R_m_[68] and shows the highest lower bound of recombination events. The accuracy of R_m_ and R_h_ to estimate the lower bound of recombination events has not been tested on sequences subjected to low and high heterogeneous mutation rates. If numerous recombination events occurred during the evolutionary history of *Pokey* elements, we may need sophisticated phylogenetic tools and analyses, such as the Ancestral Recombination Graph, to deepen our understanding of their evolutionary history.

The recombination rate of *Pokey* sequences estimated in our survey is high (3.02 × 10^-2^ events/site/generation) but comparable to the value (2.0 to 6.0 × 10^-2^ events/generation) calculated by McTaggart *et al*. [65] based on changes in the relative frequency of rDNA length variants in four apomictically-propagated *Daphnia obtusa* lines over 90 generations. To our knowledge, this is the first time that the rate of recombination has been explicitly estimated for a DNA transposon. This estimate has to be viewed with caution as some of the assumptions of the algorithm used, such as the absence of gene conversion and interference, and the selective neutrality of recombination events, may be violated. Even so, R_h_ and R_m_ estimates lead to the conclusion that the recombination rate is high between *Pokey* elements. Such a high rate of recombination between *Pokey* elements may explain why no significant differences were found between the amount of variation in 28S rRNA genes with or without *Pokey* insertions in cyclic and obligately asexual *D. pulex* isolates [69] as recombination may take place between the *Pokey* elements as well as the 28S rRNA genes themselves.

### Effects of recombination on evolution of *Pokey* elements

Numerous studies have focused on the relationship between recombination rates in host genomes and TE distributions [70-73] or dynamics [37,41,74]. The distribution and abundance of TEs may be influenced by selective forces [75-77], the stochastic process of mutation [78] and/or the availability of insertion sites. One theoretical model based on the action of selection posits that the impact of recombination events between TEs inserted in non-homologous loci is deleterious (that is, ectopic exchange model) and, therefore, insertions will accumulate in regions of the genome with low rates of recombination [77]. However, empirical data from sequenced genomes show that the relationship between TE density and recombination rate depends on the organism and the TE class or family [4,39,71,79-82].

Recombination between TE copies of the same family has been reported before [83-87], but the significance of this for TEs in general is rarely discussed. Our survey focused on variation in an approximately 1,600 bp sequence. This dataset does not include the TIRs known to be essential for transposition or the IGS sequences upstream of the transposase gene that may influence expression of the transposase [39]. If the TIRs or transposase gene have diverged in different species and recombination events produce mosaic sequences, the transposase may not be able to recognize TIRs with which it has not co-evolved. Thus, we might expect that many recombination events between divergent TE alleles will inactivate their mosaic product. Alternatively, Schaack *et al*. [74] recently suggested that TEs might benefit from recombination in a similar manner to the host, by generating new variants that are able to evade sequence-specific host suppression machinery. Recombination between different insertions could bring together independent, beneficial nucleotide substitutions that may then be favored by intragenomic selection and result in an increase in copy number of the new, recombinant insertion.

Fragments of the *Pokey* transposase gene have different evolutionary histories. Moreover, non-synonymous polymorphism coupled with recombination has produced a substantial level of *Pokey* variability at the aa level (Table 1, Additional file 2). This variability is primarily seen in exon 2, which is thought to contain a nuclear localization signal and a sequence similar to a Plant Homeo Domain or cysteine-rich zinc finger that is possibly involved in chromatin or protein-protein interactions [40,88,89]. For example, the fourth cysteine residue of the putative zinc finger is replaced either by a serine or a tyrosine residue in 3 of the 40 different aa sequences we analyzed (see Additional file 2). This variation might translate to flexibility in target site interactions or changes to dimerization dynamics during the formation of the transposition complex, which could have implications for mobility and transposition efficiency.

DNA transposons in *Caenorhabditis elegans* are located preferentially in recombination hotspot regions whereas retrotransposons are not, and Duret *et al*. [71] suggested a role for recombination in the transposition process. Glass *et al*. [69] did find indirect evidence that *Pokey* insertions in rDNA could spread through unequal crossing-over, so perhaps there is some credence to this suggestion. In addition to *Pokey* in *Daphnia,* the retrotransposons *R1* and *R2* in *Drosophila* and numerous other arthropods are also found inserted into rDNA. Using the ectopic exchange model as a framework, Zhang *et al*. [90] demonstrated that eukaryotic hosts may tolerate a high load of retrotransposable elements in their rDNA because they generally have many more rDNA copies than the minimum required for rRNA synthesis. The location of *Pokey* elements in rDNA may be advantageous for both element and host, as frequent recombination between different copies located in these loci may increase the efficiency of intragenomic selection, and insertions may be only mildly harmful due to the multi-copy nature of rDNA. *Pokey* haplotype 2 is a recombinant allele (Figures 1, Table 2) that was found in numerous hybrid and non-hybrid *Daphnia* isolates having different origins and occupying different locations and ecosystems (Table 3). This might be such a successful haplotype due to the amalgamation, through recombination, of separately beneficial mutations into a single lineage that was then able to spread to different populations and species within the *D. pulex* complex.

## Conclusions

Most of the hybrid isolates from the *D. pulex* complex analyzed in this study carry *Pokey* sequences inherited from putative parental species or mosaic sequences produced by recombination between parental types. In addition, recombination may play an important role in generating life history variation in this TE. Future studies should test the activity of recombinant *Pokey* alleles to assess if recombination events have had a significant effect on their transposition capacity. Additionally, testing for recombinants among the other families of TEs found within *D. pulex* would be useful to determine whether rates of recombination among TEs depend in part on the region of the genome they inhabit or on other properties such as mode of transposition. Moreover, the possible beneficial effects of recombination on TE-level evolution in general should be investigated more thoroughly by estimating rates of recombination for different TEs in various genomes.

## Abbreviations

Aa: amino acid;Bp: base pair;BSA: bovine serum albumin;GARD: Genetic Algorithm for Recombination Detection;IGS: intergenic spacer;mtDNA: mitochondrial DNA;PCR: polymerase chain reaction;RFLP: restriction fragment length polymorphism;SINEs: short interspersed elements;TEs: transposable elements;TIRs: terminal inverted repeats

## Competing interests

The authors declare that they have no competing interests.

## Authors’ contributions

RV designed the project and wrote the manuscript. RV and TAE planned the analyses. RV, TAE and PDP conducted the analyses. All authors analyzed the data and contributed to the writing and editing of the manuscript. All authors approved the final manuscript.

## Supplementary Material

Additional file 1 **Description of *Daphnia *isolates included in this study.** The labels of the isolates are composites of their characteristics. The first two letters represent the mitochondrial haplotypes (AR = *D. arenata*, EPC = European *D. pulicaria*, EPX = European *D. pulex*, MI = *D. middendorffiana*, PC = *D. pulicaria*, PX = *D. pulex*, TE = *D. tenebrosa*) followed by the ploidy level (2 or 3), a 2 letter country or state/province code and the isolate number. Sequences marked with an asterisk (*) were obtained from a RT-PCR product. Accession numbers refer to *Pokey* sequences amplified from *Daphnia* isolates. Click here for file

Additional file 2 **Amino acid polymorphism in 40 unique partial *Pokey *transposase sequences.** The sequences were derived from 53 *Pokey* elements obtained from members of the *Daphnia pulex* complex. Numbers at each position represent the number of sequences that carry this amino acid. Not all values sum to 40 as indels occurred in some sequences. The pink bar represents the amino sequence coded by the partial exon 1. The blue bar represents the amino sequence coded by exon 2. The dark blue regions with greek numbers represent estimated regions of recombination identified using maximum chi-square analysis ( Additional file 3). Click here for file

Additional file 3 **Recombination events in 53 partial *Pokey *sequences.** Recombination events were estimated using the maximum chi-square method. Click here for file

Additional file 4 **Groups of recombinant *Pokey *sequences.** The groups are based on phylogenetic network analysis (Figure 2) and recombination breakpoint analyses (Table 2). Click here for file

Additional file 5 **One possible ancestral *Pokey *recombination graph.** The graph was constructed from 40 different *Pokey* sequences from members of the *Daphnia pulex* complex and shows 89 recombination events. Ovals at the bottom of the graph represent *Pokey* alleles. Ovals with a trifurcation represent putative recombination events. Colors represent species or hybrid state of isolates: red = *D. pulex*, light blue = *D. arenata*, grey = *D. tenebrosa*, black = European *D. pulex*, dark blue = European *D. pulicaria*, green = *D. pulicaria*, orange = putative *pulex-pulicaria* hybrids, purple = the isolate with *D. tenebrosa* mtDNA and a *D. pulex* nuclear genome. Red lines represent the evolutionary history of the *Pokey* allele TE3-MB-4_12. Click here for file

Additional file 6 **Phylogenetic network of 58 partial *Pokey *sequences.** The network was constructed using the NeighborNet algorithm. Sequences named CO_Scaffold_xxx were obtained from the v1.1 genome sequence of *Daphnia pulex* [ 36]. Colors represent species or hybrid state of isolates: red = *D. pulex*, light blue = *D. arenata*, grey = *D. tenebrosa*, black = European *D. pulex*, dark blue = European *D. pulicaria*, green = *D. pulicaria*, orange = putative *pulex-pulicaria* hybrids, purple = the isolate with *D. tenebrosa* mtDNA and a *D. pulex* nuclear genome. Click here for file

Additional file 7 ***Pokey *RFLP haplotypes amplified from 41 isolates of the *Daphnia pulex *complex.** Location codes are defined in Additional file 1. A one letter code allows differentiation of geographical regions in the same state or province: C = Churchill, MB, CAN; W = Winnipeg, MB, CAN; K = Kuujjuarapik, QC, CAN; M = Metis, QC, CAN; S = Sainte-Foy, QC, CAN. Taxonomic codes are as follows: EPC = European *D. pulicaria*; PC = *D. pulicaria*; PX = *D. pulex*; TE = *D. tenebrosa*; Hyb= pulex-pulicaria hybrid; Int = introgressed isolate. Click here for file

## References

[B1] Feschotte C Pritham EJ DNA transposons and the evolution of eukaryotic genomes Annu Rev Genet 2007 41 331 368 10.1146/annurev.genet.40.110405.090448 18076328 PMC2167627

[B2] Shigenobu S Watanabe H Hattori M Sakaki Y Ishikawa H Genome sequence of the endocellular bacterial symbiont of aphids *Buchnera sp* APS. Nature 2000 407 81 86 10.1038/35024074 10993077

[B3] Pidpala OV Yatsishina A Lukash L Human mobile genetic elements: structure, distribution and functional role Cytol Genet 2008 42 420 430 10.3103/S009545270806011X 19253758

[B4] Eickbush DG Eickbush TH Vertical transmission of the retrotransposable elements R1 and R2 during the evolution of the *Drosophila melanogaster* species subgroup Genetics 1995 139 671 684 7713424 10.1093/genetics/139.2.671 PMC1206373

[B5] Robertson HM Lampe DJ Recent horizontal transfer of a mariner transposable element among and between Diptera and Neuroptera Mol Biol Evol 1995 12 850 862 7476131 10.1093/oxfordjournals.molbev.a040262

[B6] Gonzalez P Lessios HA Evolution of sea urchin retroviral-like (SURL) elements: evidence from 40 echinoid species Mol Biol Evol 1999 16 938 952 10.1093/oxfordjournals.molbev.a026183 10406111

[B7] Penton EH Crease TJ Evolution of the transposable element *Pokey* in the ribosomal DNA of species in the subgenus *Daphnia* (Crustacea: Cladocera) Mol Biol Evol 2004 21 1727 1739 10.1093/molbev/msh189 15201395

[B8] Shedlock AM Okada N SINE insertions: powerful tools for molecular systematics Bioessays 2000 22 148 160 10.1002/(SICI)1521-1878(200002)22:2<148::AID-BIES6>3.0.CO;2-Z 10655034

[B9] Capy P Anxolabéhère D Langin T The strange phylogenies of transposable elements: are horizontal transfers the only explanation? Trends Genet 1994 10 7 12 10.1016/0168-9525(94)90012-4 8146915

[B10] Loreto ELS Carareto CMA Capy P Revisiting horizontal transfer of transposable elements in *Drosophila* Heredity 2008 100 545 554 10.1038/sj.hdy.6801094 18431403

[B11] Gilbert C Schaack S Pace Ii JK Brindley PJ Feschotte C A role for host-parasite interactions in the horizontal transfer of transposons across phyla Nature 2010 464 1347 1350 10.1038/nature08939 20428170 PMC3004126

[B12] Silva J Loreto E Clark J Factors that affect the horizontal transfer of transposable elements Curr Issues Mol Biol 2004 6 57 71 14632259

[B13] Yoshiyama M Tu Z Kainoh Y Honda H Shono T Kimura K Possible horizontal transfer of a transposable element from host to parasitoid Mol Biol Evol 2001 18 1952 1958 10.1093/oxfordjournals.molbev.a003735 11557800

[B14] Hebert PDN Obligate asexuality in *Daphnia* Am Nat 1981 117 784 789 10.1086/283761

[B15] Hebert PDN Crease T Clonal diversity in populations of *Daphnia pulex* reproducing by obligate parthenogenesis Heredity 1983 51 353 369 10.1038/hdy.1983.40

[B16] Innes DJ Schwartz SS Hebert PDN Genotypic diversity and variation in mode of reproduction among populations in the *Daphnia pulex* group Heredity 1986 57 345 355 10.1038/hdy.1986.134

[B17] Innes DJ Fox CJ Winsor GL Avoiding the cost of males in obligately asexual *Daphnia pulex* (Leydig) Proc R Soc Lond B Biol Sci 2000 267 991 997 10.1098/rspb.2000.1101 PMC1690637 10874748

[B18] Hebert P Finston T Macrogeographic patterns of breeding system diversity in the Daphnia pulex group from the United States and Mexico Heredity 2001 87 153 161 10.1046/j.1365-2540.2001.00885.x 11703505

[B19] Hebert PDN The Daphnia of North America: an illustrated fauna 1995 Guelph, Ontario: University of Guelph

[B20] Colbourne JK Crease TJ Weider LJ Hebert PDN Dufresne F Hobaek A Phylogenetics and evolution of a circumarctic species complex (Cladocera: *Daphnia pulex*) Biol J Linn Soc 1998 65 347 365

[B21] Marková S Dufresne F Rees DJ Cerný M Kotlík P Cryptic intercontinental colonization in water fleas *Daphnia pulicaria* inferred from phylogenetic analysis of mitochondrial DNA variation Mol Phylogenet Evol 2007 44 42 52 10.1016/j.ympev.2006.12.025 17292634

[B22] Vergilino R Markova S Ventura M Manca M Dufresne F Reticulate evolution of the *Daphnia pulex* complex as revealed by nuclear markers Mol Ecol 2011 20 1191 1207 10.1111/j.1365-294X.2011.05004.x 21294799

[B23] Crease TJ Stanton DJ Hebert PDN Polyphyletic origins of asexuality in *Daphnia pulex.* II. Mitochondrial-DNA variation Evolution 1989 43 1016 1026 10.2307/2409582 28564160

[B24] Hebert PDN Beaton MJ Schwartz SS Stanton DJ Polyphyletic origins of asexuality in *Daphnia pulex.* I. Breeding-system variation and levels of clonal diversity Evolution 1989 43 1004 1015 10.2307/2409581 28564164

[B25] Hebert PDN Schwartz SS Ward RD Finston TL Macrogeographic patterns of breeding system diversity in the *Daphnia pulex* group. I. Breeding systems of Canadian populations Heredity 1993 70 148 161 10.1038/hdy.1993.24 11703505

[B26] Innes DJ Hebert PDN The origin and genetic basis of obligate parthenogenesis in *Daphnia pulex* Evolution 1988 42 1024 1035 10.2307/2408918 28581165

[B27] Heier CR Dudycha JL Ecological speciation in a cyclic parthenogen: sexual capability of experimental hybrids between *Daphnia pulex* and *Daphnia pulicaria* Limnol Oceanogr 2009 54 492 502 10.4319/lo.2009.54.2.0492

[B28] Cristescu ME Constantin A Bock DG Caceres CE Crease TJ Speciation with gene flow and the genetics of habitat transitions Mol Ecol 2012 21 1411 1422 10.1111/j.1365-294X.2011.05465.x 22269101

[B29] Beaton MJ Hebert PDN Geographical parthenogenesis and polyploidy in *Daphnia pulex* Am Nat 1988 132 837 845 10.1086/284892

[B30] Dufresne F Hebert PDN Hybridization and origins of polyploidy Proc R Soc Lond B Biol Sci 1994 258 141 146 10.1098/rspb.1994.0154

[B31] Dufresne F Hebert PDN Polyploidy and clonal diversity in an arctic cladoceran Heredity 1995 75 45 53 10.1038/hdy.1995.102

[B32] Dufresne F Hebert PDN Pleistocene glaciations and polyphyletic origins of polyploidy in an arctic cladoceran Proc R Soc Lond B Biol Sci 1997 264 201 206 10.1098/rspb.1997.0028

[B33] Weider LJ Hobaek A Hebert PDN Crease TJ Holarctic phylogeography of an asexual species complex - II. Allozymic variation and clonal structure in Arctic Daphnia Mol Ecol 1999 8 1 13 10.1046/j.1365-294X.1999.00522.x

[B34] Ambrose C Crease T Evolution of the nuclear ribosomal DNA intergenic spacer in four species of the *Daphnia pulex* complex BMC Genet 2011 12 13 21261945 10.1186/1471-2156-12-13 PMC3036644

[B35] Vergilino R Belzile C Dufresne F Genome size evolution and polyploidy in the *Daphnia pulex* complex (Cladocera: Daphniidae) Biol J Linn Soc 2009 97 68 79 10.1111/j.1095-8312.2008.01185.x

[B36] Colbourne JK Pfrender ME Gilbert D Thomas WK Tucker A Oakley TH Tokishita S Aerts A Arnold GJ Basu MK Bauer DJ Cáceres CE Carmel L Casola C Choi JH Detter JC Dong Q Dusheyko S Eads BD Fröhlich T Geiler-Samerotte KA Gerlach D Hatcher P Jogdeo S Krijgsveld J Kriventseva EV Kültz D Laforsch C Lindquist E Lopez J The ecoresponsive genome of *Daphnia pulex* Science 2011 331 555 561 10.1126/science.1197761 21292972 PMC3529199

[B37] Schaack S Choi E Lynch M Pritham E DNA transposons and the role of recombination in mutation accumulation in *Daphnia pulex* Genome Biol 2010 11 R46 10.1186/gb-2010-11-4-r46 20433697 PMC2884549

[B38] Wicker T Sabot F Hua-Van A Bennetzen JL Capy P Chalhoub B Flavell A Leroy P Morgante M Panaud O Paux E SanMiguel P Schulman AH A unified classification system for eukaryotic transposable elements Nat Rev Genet 2007 8 973 982 10.1038/nrg2165 17984973

[B39] Penton EH Sullender BW Crease TJ *Pokey*, a new DNA transposon in *Daphnia* (Cladocera: Crustacea) J Mol Evol 2002 55 664 673 10.1007/s00239-002-2362-9 12486525

[B40] Elliott TA The activity and evolution of the Daphnia DNA transposon Pokey.MSc. Thesis 2011 University of Guelph, Department of Integrative Biology

[B41] Valizadeh P Crease T The association between breeding system and transposable element dynamics in *Daphnia pulex* J Mol Evol 2008 66 643 654 10.1007/s00239-008-9118-0 18491025

[B42] Sullender BW Crease TJ The behavior of a *Daphnia pulex* transposable element in cyclically and obligately parthenogenetic populations J Mol Evol 2001 53 63 69 11683324 10.1007/s002390010193

[B43] Eickbush TH Eickbush DG Finely orchestrated movements: evolution of the ribosomal RNA genes Genetics 2007 175 477 485 10.1534/genetics.107.071399 17322354 PMC1800602

[B44] Hebert PDN Crease TJ Clonal coexistence in *Daphnia pulex* (Leydig): another planktonic paradox Science 1980 207 1363 1365

[B45] Hall TA BioEdit: a user-friendly biological sequence alignment editor and analysis program for Windows 95/98/NT Nucleic Acids Symp Ser 1999 41 95 98

[B46] Bruen TC Philippe H Bryant D A simple and robust statistical test to detect the presence of recombination Genetics 2006 172 2665 2681 16489234 10.1534/genetics.105.048975 PMC1456386

[B47] Huson DH Bryant D Application of phylogenetic networks in evolutionary studies Mol Biol Evol 2006 23 254 267 16221896 10.1093/molbev/msj030

[B48] Kosakovsky Pond SL Posada D Gravenor MB Woelk CH Frost SDW GARD: a genetic algorithm for recombination detection Bioinformatics 2006 22 3096 3098 10.1093/bioinformatics/btl474 17110367

[B49] Hudson RR Kaplan NL Statistical properties of the number of recombination events in the history of a sample of dna sequences Genetics 1985 111 147 164 4029609 10.1093/genetics/111.1.147 PMC1202594

[B50] Librado P Rozas J DnaSP v5: A software for comprehensive analysis of DNA polymorphism data Bioinformatics 2009 25 1451 1452 10.1093/bioinformatics/btp187 19346325

[B51] Lyngsø R Song Y Hein J Casadio R, Myers G Minimum recombination histories by branch and bound Algorithms in Bioinformatics. Volume 3692 2005 Berlin/Heidelberg: Springer 239 250 [Lecture Notes in Computer Science.]

[B52] Song YS Hein J Constructing minimal ancestral recombination graphs J Comput Biol 2005 12 147 169 10.1089/cmb.2005.12.147 15767774

[B53] Kuhner MK Coalescent genealogy samplers: windows into population history Trends Ecology Evol 2009 24 86 93 10.1016/j.tree.2008.09.007 PMC4714702 19101058

[B54] Tamura K Peterson D Peterson N Stecher G Nei M Kumar S MEGA5: Molecular Evolutionary Genetics Analysis using maximum likelihood, evolutionary distance, and maximum parsimony methods Mol Biol Evol 2011 28 2731 2739 10.1093/molbev/msr121 21546353 PMC3203626

[B55] Geyer CJ Estimating normalizing constants and reweighting mixtures in Markov chain Monte Carlo. Technical Report No. 568 1991 Minneapolis: School of Statistics, University of Minnesota http://purl.umn.edu/58433

[B56] Posada D Evaluation of methods for detecting recombination from DNA sequences: empirical data Mol Biol Evol 2002 19 708 717 10.1093/oxfordjournals.molbev.a004129 11961104

[B57] Posada D Crandall KA Evaluation of methods for detecting recombination from DNA sequences: computer simulations Proc Natl Acad Sci U S A 2001 98 13757 13762 10.1073/pnas.241370698 11717435 PMC61114

[B58] Posada D Crandall KA Holmes EC Recombination in evolutionary genomics Annu Rev Genet 2002 36 75 97 10.1146/annurev.genet.36.040202.111115 12429687

[B59] Graham J McNeney B Seillier-Moiseiwitsch F Stepwise detection of recombination breakpoints in sequence alignments Bioinformatics 2005 21 589 595 10.1093/bioinformatics/bti040 15388518

[B60] Maynard Smith J Analyzing the mosaic structure of genes J Mol Evol 1992 34 126 129 1556748 10.1007/BF00182389

[B61] Saitou N Nei M The neighbor-joining method: a new method for reconstructing phylogenetic trees Mol Biol Evol 1987 4 406 425 3447015 10.1093/oxfordjournals.molbev.a040454

[B62] Song YS Hein J Benson G, Page R Parsimonious reconstruction of sequence evolution and haplotype blocks Algorithms in Bioinformatics. Volume 2812 2003 Berlin/Heidelberg: Springer 287 302 Lecture Notes in Computer Science

[B63] Qiu X Wu L Huang H McDonel PE Palumbo AV Tiedje JM Zhou J Evaluation of PCR-generated chimeras, mutations, and heteroduplexes with 16S rRNA gene-based cloning Appl Environ Microbiol 2001 67 880 887 10.1128/AEM.67.2.880-887.2001 11157258 PMC92662

[B64] Crease T Floyd R Cristescu M Innes D Evolutionary factors affecting lactate dehydrogenase A and B variation in the *Daphnia pulex* species complex BMC Evol Biol 2011 11 212 10.1186/1471-2148-11-212 21767386 PMC3231769

[B65] McTaggart SJ Dudycha JL Omilian A Crease TJ Rates of recombination in the ribosomal DNA of apomictically propagated *Daphnia obtusa* lines Genetics 2007 175 311 320 17110499 10.1534/genetics.105.050229 PMC1775004

[B66] Omilian AR Lynch M Patterns of intraspecific DNA variation in the *Daphnia* nuclear genome Genetics 2009 182 325 336 10.1534/genetics.108.099549 19304589 PMC2674828

[B67] Bafna V Bansal V The number of recombination events in a sample history: conflict graph and lower bounds IEEE/ACM Trans Comput Biol Bioinformatics 2004 1 78 90 10.1109/TCBB.2004.23 17048383

[B68] Myers SR Griffiths RC Bounds on the minimum number of recombination events in a sample history Genetics 2003 163 375 394 12586723 10.1093/genetics/163.1.375 PMC1462432

[B69] Glass SK Moszczynska A Crease TJ The effect of transposon *Pokey* insertions on sequence variation in the 28S rRNA gene of *Daphnia pulex* Genome 2008 51 988 1000 10.1139/G08-092 19088812

[B70] Carr M Soloway J Robinson T Brookfield J Mechanisms regulating the copy numbers of six LTR retrotransposons in the genome of *Drosophila melanogaster* Chromosoma 2002 110 511 518 10.1007/s00412-001-0174-0 12068968

[B71] Duret L Marais G Biemont C Transposons but not retrotransposons are located preferentially in regions of high recombination rate in *Caenorhabditis elegans* Genetics 2000 156 1661 1669 11102365 10.1093/genetics/156.4.1661 PMC1461346

[B72] Langley CH Montgomery E Hudson R Kaplan N Charlesworth B On the role of unequal exchange in the containment of transposable element copy number Genet Res 1988 52 223 235 10.1017/S0016672300027695 2854088

[B73] Rizzon C Martin E Marais G Duret L Segalat L Biemont C Patterns of selection against transposons inferred from the distribution of Tc1, Tc3 and Tc5 insertions in the mut-7 line of the nematode *Caenorhabditis elegans* Genetics 2003 165 1127 1135 14668370 10.1093/genetics/165.3.1127 PMC1462815

[B74] Schaack S Pritham EJ Wolf A Lynch M DNA transposon dynamics in populations of *Daphnia pulex* with and without sex Proc R Soc B Biol Sci 2010 277 2381 2387 10.1098/rspb.2009.2253 PMC2894897 20356890

[B75] Brookfield J Models of repression of transposition in P-M hybrid dysgenesis by P cytotype and by zygotically encoded repressor proteins Genetics 1991 128 471 486 1649073 10.1093/genetics/128.2.471 PMC1204483

[B76] Finnegan DJ Lindsley DL, Zimm GG Transposable elements The Genome of Drosophila melanogaster. Volume viii 1992 New York: Academic 1096 1107

[B77] Montgomery E Charlesworth B Langley CH A test for the role of natural selection in the stabilization of transposable element copy number in a population of *Drosophila melanogaster* Genet Res 1987 49 31 41 10.1017/S0016672300026707 3032743

[B78] Biémont C Population genetics of transposable DNA elements Genetica 1992 86 67 84 10.1007/BF00133712 1334919

[B79] Bartolomé C Maside X Charlesworth B On the abundance and distribution of transposable elements in the genome of *Drosophila melanogaster* Mol Biol Evol 2002 19 926 937 10.1093/oxfordjournals.molbev.a004150 12032249

[B80] Kim JM Vanguri S Boeke JD Gabriel A Voytas DF Transposable elements and genome organization: a comprehensive survey of retrotransposons revealed by the complete *Saccharomyces cerevisiae* genome sequence Genome Res 1998 8 464 478 9582191 10.1101/gr.8.5.464

[B81] Rizzon C Marais G Gouy M Biemont C Recombination rate and the distribution of transposable elements in the *Drosophila melanogaster* genome Genome Res 2002 12 400 407 11875027 10.1101/gr.210802 PMC155295

[B82] Wright SI Agrawal N Bureau TE Effects of recombination rate and gene density on transposable element distributions in *Arabidopsis thaliana* Genome Res 2003 13 1897 1903 12902382 10.1101/gr.1281503 PMC403781

[B83] Bao W Jurka J Origin and evolution of LINE-1 derived "half-L1" retrotransposons (HAL1) Gene 2010 465 9 16 10.1016/j.gene.2010.06.005 20600705 PMC2923044

[B84] Bleykasten-Grosshans C Jung PP Fritsch ES Potier S de Montigny J Souciet JL The Ty1 LTR-retrotransposon population in *Saccharomyces cerevisiae* genome: dynamics and sequence variations during mobility FEMS Yeast Res 2011 11 334 344 10.1111/j.1567-1364.2011.00721.x 21272231

[B85] Fischer SEJ Wienholds E Plasterk RHA Continuous Exchange of Sequence Information Between Dispersed Tc1 Transposons in the *Caenorhabditis elegans* Genome Genetics 2003 164 127 134 12750326 10.1093/genetics/164.1.127 PMC1462561

[B86] Jordan IK McDonald JF Evidence for the role of recombination in the regulatory evolution of *Saccharomyces cerevisiae* Ty elements J Mol Evol 1998 47 14 20 10.1007/PL00006358 9664692

[B87] Novick PA Smith JD Floumanhaft M Ray DA Boissinot S The evolution and diversity of DNA transposons in the genome of the lizard *Anolis carolinensis* Genome Biol Evol 2011 3 1 14 10.1093/gbe/evq080 21127169 PMC3014272

[B88] Keith JH Schaeper CA Fraser TS Fraser MJ Mutational analysis of highly conserved aspartate residues essential to the catalytic core of the piggyBac transposase BMC Mol Biol 2008 9 73 10.1186/1471-2199-9-73 18694512 PMC2533014

[B89] Mitra R Fain-Thornton J Craig NL piggyBac can bypass DNA synthesis during cut and paste transposition EMBO J 2008 27 1097 1109 10.1038/emboj.2008.41 18354502 PMC2323262

[B90] Zhang X Eickbush MT Eickbush TH Role of recombination in the long-term retention of transposable elements in rRNA gene loci Genetics 2008 180 1617 1626 10.1534/genetics.108.093716 18791229 PMC2581962

