# Supplementary material for: Evolution of a transposon in Daphnia hybrid genomes
Source: Mob DNA. 2013 Feb 6;4:7. doi: 10.1186/1759-8753-4-7 (PMC3575242; doi:10.1186/1759-8753-4-7)
Supplement: Additional file 1 — Description of Daphnia isolates included in this study. The labels of the isolates are composites of their characteristics. The first two letters represent the mitochondrial haplotypes (AR = D. arenata, EPC = European D. pulicaria, EPX = European D. pulex, MI = D. middendorffiana, PC = D. pulicaria, PX = D. pulex, TE = D. tenebrosa) followed by the ploidy level (2 or 3), a 2 letter country or state/province code and the isolate number. Sequences marked with an asterisk (*) were obtained from a RT-PCR product. Accession numbers refer to Pokey sequences amplified from Daphnia isolates. [file 1759-8753-4-7-S1.pdf]

Additional File 1. Description of *Daphnia* isolates included in this study.

The labels of the isolates are composites of their characteristics. The first two letters represent the mitochondrial haplotypes (AR = *D. arenata*, EPC = European *D. pulicaria*, EPX = European *D. pulex*, MI = *D. middendorffiana*, PC = *D. pulicaria*, PX = *D. pulex*, TE= *D. tenebrosa*) followed by the ploidy level (2 or 3), a 2 letter country or state/province code and the isolate number. Sequences marked with an asterisk (*) were obtained from a RT-PCR product. Accession numbers refer to *Pokey* sequences amplified from *Daphnia* isolates.

Isolates	mitochondrial haplotype	Ploidy level	*Ldh* genotype	Accession number	Geographic location
PX2-MB-1	North-American *D. pulex*	2x	SS	/	Churchill, MB, CAN
PX2-MB-2	North-American *D. pulex*	2x	SS	/	Churchill, MB, CAN
PX2-ON-3	North-American *D. pulex*	2x	SS	/	Windsor, ON, CAN
PX2-ON-4	North-American *D. pulex*	2x	SS	/	Windsor, ON, CAN
PX2-ON-9	North-American *D. pulex*	2X	SS	[JX848694]	Windsor, ON, CAN
PX2-MB-3	North-American *D. pulex*	2x	SF	[JX838828]	Churchill, MB, CAN
PX2-ON-2	North-American *D. pulex*	2x	SF	/	Windsor, ON, CAN
PX2-ON-10	North-American *D. pulex*	2x	SF	/	Windsor, ON, CAN
PX2-QC-5	North-American *D. pulex*	2x	SF	/	Ste-Foy, QC, CAN
PX2-QC-6	North-American *D. pulex*	2x	SF	/	Ste-Foy, QC, CAN
PX2-MI-11	North-American *D. pulex*	2x	SF	/	MI, USA
PX2-QC-8	North-American *D. pulex*	2x	SF	[JX838839 to JX838845]	Kuujjarapik, QC, CAN
PX2-QC-9	North-American *D. pulex*	2x	SF	/	Métis, QC, CAN
PX2-QC-12	North-American *D. pulex*	2x	SF	/	Métis, QC, CAN
PX3-QC-1	North-American *D. pulex*	3x	SF	[JX838848 to JX838850]	Kuujjarapik, QC, CAN
PX3-QC-2	North-American *D. pulex*	3x	SF	/	Kuujjarapik, QC, CAN

PC3-QC-1	Eastern *D. pulicaria*	3x	SF	[JX838851]	Kuujjarapik, QC, CAN
PC3-QC-2	Eastern *D. pulicaria*	3x	SF	/	Kuujjarapik, QC, CAN
PC3-QC-3	Eastern *D. pulicaria*	3x	SF	[JX838846, JX838847]	Kuujjarapik, QC, CAN
PX2-IL-1	North-American *D. pulex*	2x	FF	/	Vermillion County, IL, USA
PX2-IL-2	North-American *D. pulex*	2x	FF	/	Vermillion County, IL, USA
PX2-IL-3	North-American *D. pulex*	2x	FF	/	Vermillion County, IL, USA
PX2-IL-4	North-American *D. pulex*	2x	FF	/	Vermillion County, IL, USA
PC3-MB-4	Polar *D. pulicaria*	3x	SF	/	Churchill, MB, CAN
PC3-MB-5	Polar *D. pulicaria*	3x	SF	[JX838811, JX838812]	Churchill, MB, CAN
PC2-IN-1	Western *D. pulicaria*	2x	FF	/	IN, USA
PC2-IN-2	Western *D. pulicaria*	2x	FF	/	IN, USA
PC2-MB-3	Western *D. pulicaria*	2x	FF	/	Winnipeg, MB, CAN
PC2-QC-4	Western *D. pulicaria*	2x	FF	[JX838808 to JX838810]	Kuujjarapik, QC, CAN
PC3-MB-6	Western *D. pulicaria*	3x	SF	/	Churchill, MB, CAN
PC3-MB-7	Western *D. pulicaria*	3x	SF	/	Kuujjarapik, QC, CAN
MI3-MB-1	*D. Middendorffiana s.s.*	3x	SF	/	Churchill, MB, CAN
MI3-MB-2	*D. Middendorffiana s.s.*	3x	SF	[JX838813]	Churchill, MB, CAN
TE2-MB-1	*D. tenebrosa*	2x	UN	[JX838832, JX838833]	Churchill, MB, CAN

TE2-MB-2	*D. tenebrosa*	2x	UN	[JX838836]	Churchill, MB, CAN
TE2-MB-3	*D. tenebrosa*	2x	UN	[JX838837, JX838838]	Churchill, MB, CAN
TE3-MB-1	*D. tenebrosa*	3x	UN	[JX838819 to JX838822]	Churchill, MB, CAN
TE3-MB-2	*D. tenebrosa*	3x	UN	[JX838823 to JX838827]	Churchill, MB, CAN
TE3-MB-3	*D. tenebrosa*	3x/4x	UN	[JX838829 to JX838831]	Churchill, MB, CAN
TE3-MB-4	*D. tenebrosa*	3x	SS	[JX838814 to JX838818]	Churchill, MB, CAN
EPC2-CZ-1	European *D. pulicaria*	2x	UN	[JX838834]	Lowland, CZE
EPC2-SP-2	European *D. pulicaria*	2x	UN	[JX838835]	Catalonia, ESP
EPC2-DE-3	European *D. pulicaria*	2x	UN	[AY630582.1]	Grosser Binnense, DEU
EPX2-DE-1	European *D. pulex*	2x	UN	[AY630583.1]	Grebin, DEU
EPX2-DE-2	European *D. pulex*	2x	UN	[AY630584.1]	Malente, DEU
PC2-SK-5	*D. pulicaria*	2x	FF	[AY630577.1, AY630578.1]	Humbolt Lake, SK, CAN
PX2-IL-5	North-American *D. pulex*	2x	SS	[AY630581.1]	Vermillion County, IL, USA
PX2-IN-6	North-American *D. pulex*	2x	SS		Warren County, IN, USA
PX2-ON-7	North-American *D. pulex*	2x	SS	[AY630579.1]	Essex County, ON, CAN
PX2-QC-13	North-American *D. pulex*	2x	SS	[AY630580.1]	La Tuque, QC, CAN
AR2-OR-1	North-American *D. pulex*	2x	UN	[AY630585.1]	Florence, OR, USA
