# Supplementary material for: Evolution of a transposon in Daphnia hybrid genomes
Source: Mob DNA. 2013 Feb 6;4:7. doi: 10.1186/1759-8753-4-7 (PMC3575242; doi:10.1186/1759-8753-4-7)
Supplement: Additional file 2 — Amino acid polymorphism in 40 unique partial Pokey transposase sequences. The sequences were derived from 53 Pokey elements obtained from members of the Daphnia pulex complex. Numbers at each position represent the number of sequences that carry this amino acid. Not all values sum to 40 as indels occurred in some sequences. The pink bar represents the amino sequence coded by the partial exon 1. The blue bar represents the amino sequence coded by exon 2. The dark blue regions with greek numbers represent estimated regions of recombination identified using maximum chi-square analysis ( Additional file 3). [file 1759-8753-4-7-S2.pdf]

Additional File 2. **Amino acid polymorphism in 40 unique partial *Pokey* transposase sequences.**

The sequences were derived from 53 *Pokey* elements obtained from members of the *Daphnia pulex* complex.Numbers at each position represent the number of sequences that carry this amino acid. Not all values sum to 40 as indels occurred in some sequences. The pink bar represents the amino sequence coded by the partial exon 1. The blue bar represents the amino sequence coded by exon 2. The dark blue regions with greek numbers represent estimated regions of recombination identified using maximum chi-square analysis (Additional File 3).

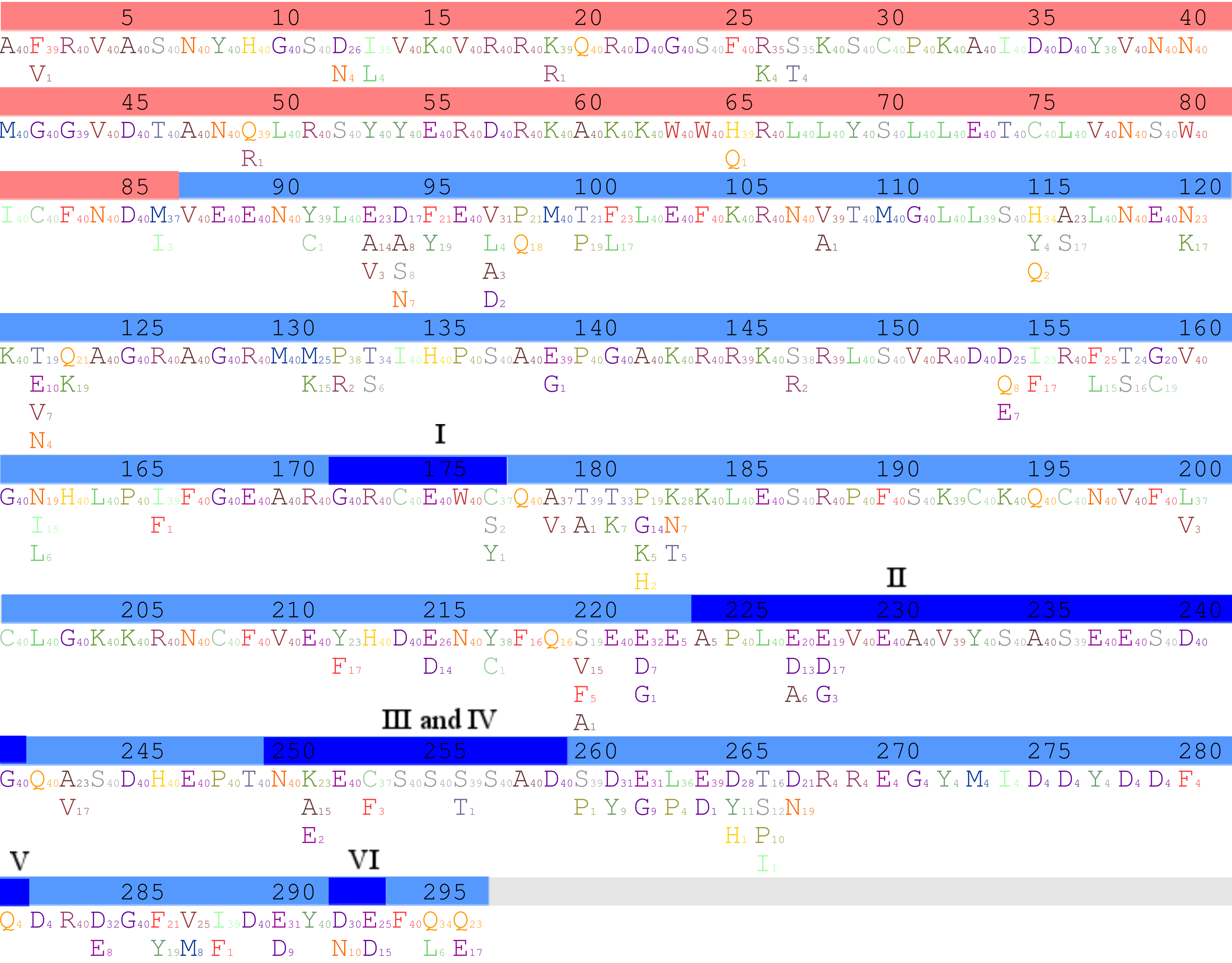

5 10 15 20 25 30 35 40
A40 F39 R40 V40 A40 S40 N40 Y40 H40 G40 S40 D26 I35 V40 K40 V40 R40 R40 K39 Q40 R40 D40 G40 S40 F40 R35 S35 K40 S40 C40 P40 K40 A40 I40 D40 D40 Y38 V40 N40 N40
V1 N4 L4 R1 K4 T4
45 50 55 60 65 70 75 80
M40 G40 G39 V40 D40 T40 A40 N40 Q39 L40 R40 S40 Y40 Y40 E40 R40 D40 R40 K40 A40 K40 K40 W40 W40 H39 R40 L40 L40 Y40 S40 L40 L40 E40 T40 C40 L40 V40 N40 S40 W40
R1 Q1
85 90 95 100 105 110 115 120
I40 C40 F40 N40 D40 M37 V40 E40 E40 N40 Y39 L40 E23 D17 F21 E40 V31 P21 M40 T21 F23 L40 E40 F40 K40 R40 N40 V39 T40 M40 G40 L40 L39 S40 H34 A23 L40 N40 E40 N23
I3 C1 A14 A8 Y19 L4 Q18 P19 L17 A1 Y4 S17 K17
V3 S8 A3 Q2
N7 D2
125 130 135 140 145 150 155 160
K40 T19 Q21 A40 G40 R40 A40 G40 R40 M40 M25 P38 T34 I40 H40 P40 S40 A40 E39 P40 G40 A40 K40 R40 R39 K40 S38 R39 L40 S40 V40 R40 D40 D25 I23 R40 F25 T24 G20 V40
E10 K19 K15 R2 S6 G1 R2 Q8 F17 L15 S16 C19
V7 E7
N4
I
165 170 175 180 185 190 195 200
G40 N19 H40 L40 P40 I39 F40 G40 E40 A40 R40 G40 R40 C40 E40 W40 C37 Q40 A37 T39 T33 P19 K28 K40 L40 E40 S40 R40 P40 F40 S40 K39 C40 K40 Q40 C40 N40 V40 F40 L37
I15 F1 S2 V3 A1 K7 G14 N7 V3
L6 Y1 K5 T5
H2
II
205 210 215 220 225 230 235 240
C40 L40 G40 K40 K40 R40 N40 C40 F40 V40 E40 Y23 H40 D40 E26 N40 Y38 F16 Q16 S19 E40 E32 E5 A5 P40 L40 E20 E19 V40 E40 A40 V39 Y40 S40 A40 S39 E40 E40 S40 D40
F17 D14 C1 V15 D7 D13 D17
F5 G1 A6 G3
A1
III and IV
245 250 255 260 265 270 275 280
G40 Q40 A23 S40 D40 H40 E40 P40 T40 N40 K23 E40 C37 S40 S40 S39 S40 A40 D40 S39 D31 E31 L36 E39 D28 T16 D21 R4 R4 E4 G4 Y4 M4 I4 D4 D4 Y4 D4 D4 F4
V17 A15 F3 T1 P1 Y9 G9 P4 D1 Y11 S12 N19
E2 H1 P10
I1
V VI
285 290 295
Q4 D4 R40 D32 G40 F21 V25 I39 D40 E31 Y40 D30 E25 F40 Q34 Q23
E8 Y19 M8 F1 D9 N10 D15 L6 E17
