# Supplementary material for: Evolution of a transposon in Daphnia hybrid genomes
Source: Mob DNA. 2013 Feb 6;4:7. doi: 10.1186/1759-8753-4-7 (PMC3575242; doi:10.1186/1759-8753-4-7)
Supplement: Additional file 3 — Recombination events in 53 partial Pokey sequences. Recombination events were estimated using the maximum chi-square method. [file 1759-8753-4-7-S3.pdf]

Additional File 3. Recombination events in 53 partial Pokey sequences.
Recombination events were estimated using the maximum chi-square method.

Event assigned	Grouped event	Effective sites	Group pair	Half-window sizes for which event has been detected (expected sites)
A	**I**	592-607	c : a	70 (547-717)
				80 (547-549, 557, 608, 614-711)
				90 (556-737)
				100 (663-707, 730, 737-756, 801-804)
B		592-607	a : PX2-QC-8_29	70 (488-511)
				80 (547, 555-556, 567-602, 610)
C		592-607	TE3-MB-4_9 : i	70 (532-534)
D		592-607	h : a	70 (516-531)
E		592-607	a : g	80 (531-545)
F	**II**	748-800	i : b	70 (722-768)
				80 (717-795)
				90 (738-853)
G		748-800	i : EPC2-SP-2_1	90 (759-779)
H	**III**	815-856	i : PX3-QC-1_20	70 (779-795)
				100 (816-864)
I	**IV**	825-856	i : c	70 (800-873)
				80 (800-933)
				90 (871-956)
				100 (974-979)
J		825-856	a : b	90 (857-868)
K		825-856	a : EPC2-SP-2_1	90 (857-868)
L		825-856	h : EPC2-SP-2_1	100 (957)
M	**V**	917-920	h : c	70 (874-927)
				90 (957-970)
N		917-920	h : a	70 (929-931)
				100 (957-970)
O	**VI**	952-957	c : g	80 (934-946)
				90 (974)
P	**VII**	1038-1073	a : g	70 (933-1002, 1008)
				80 (951-1002)
				90 (979-982)
Q		1038-1073	TE3-MB-4_9 : g	70 (1003, 1009-1013)

			80 (1003-1013)
			90 (987-1023)
R	1038-1073	TE3-MB-1_9 : PX2-MB-8_1	70 (1097-1118, 1165)
S	1038-1073	TE3-MB-1_9 : PX2-MB-8_29	70 (1020-1118, 1165)
			80 (1020-1080)
T	1038-1073	TE3-MB-1_9 : f	70 (1158-1160)
