# Supplementary material for: Evolution of a transposon in Daphnia hybrid genomes
Source: Mob DNA. 2013 Feb 6;4:7. doi: 10.1186/1759-8753-4-7 (PMC3575242; doi:10.1186/1759-8753-4-7)
Supplement: Additional file 4 — Groups of recombinant Pokey sequences. The groups are based on phylogenetic network analysis (Figure 2) and recombination breakpoint analyses (Table 2). [file 1759-8753-4-7-S4.pdf]

Additional File 4. Groups of recombinant Pokey sequences. The groups are based on phylogenetic network analysis (Figure 2) and recombination breakpoint analyses (Table 2).

Group	Sequences	Genbank number
**a**	TE3-MB-4_6	JX838814
	TE3-MB-4_12	JX838816
	TE3-MB-4_13	JX838817
	TE3-MB-1_17	JX838818
	TE3-MB-4_9	JX838815
**b**	PC3-QC-3_5	JX838846
	PX2-QC-8_3	JX838841
	TE2-MB-3_3	JX838837
	TE2-MB-3_20	JX838838
	TE3-MB-1_14	JX838821
	TE3-MB-2_13	JX838825
	EPC2-DE-3_1	AY630582.1
	EPC2-SP-2_1	JX838835
**c**	PC3-MB-5_15	JX838811
	PC3-MB-5_17	JX838812
	MI3-MB-2_1	JX838813
**d**	TE2-MB-1_18	JX838833
	TE3-MB-1_2	JX838820
	TE3-MB-1_3	JX838819
	TE3-MB-1_19	JX838822
	TE3-MB-3_1	JX838829
	TE3-MB-3_4	JX838830
	TE3-MB-3_25	JX838831
	TE3-MB-2_10	JX838824
	TE3-MB-2_14	JX838823
	TE3-MB-2_19	JX838826
	TE3-MB-2_21	JX838827
**e**	EPX2-DE-1	AY630583.1
	EPX2-DE-2	AY630584.1
**f**	PC2-SK-5_1	AY630577.1
	EPC2-CZ-1_1	JX838834
	TE2-MB-1_8	JX838832
	PC3-QC-3_17	JX838847
	PX2-QC-8_1	JX838839
	PX2-QC-8_29	JX838843
**g**	PC2-QC-4_1	JX838808
	PC2-QC-4_2	JX838809
	PC2-SK-5_1	AY630578.1
	PC3-QC-1_28	JX838851
	PX2-QC-8_2	JX838840
	PX2-QC-8_4	JX838845
	PX2-QC-8_28	JX838842
	PX3-QC-1_1	JX838848
	PX3-QC-1_9	JX838849
	TE2-MB-2_6	JX838836
	PX3-QC-1_20	JX838850
**h**	PX2-MB-1_18	JX838828
	PX2-US-CON	AY630581.1

**h**	PX2-MB-1_18	JX838828
	PX2-US-CON	AY630581.1
	PX2-ON-7_1	AY630579.1
	PX2-QC-13_1	AY630580.1
**i**	AR2-OR-1_1	AY630585.1
