# Supplementary material for: Evolution of a transposon in Daphnia hybrid genomes
Source: Mob DNA. 2013 Feb 6;4:7. doi: 10.1186/1759-8753-4-7 (PMC3575242; doi:10.1186/1759-8753-4-7)
Supplement: Additional file 6 — Phylogenetic network of 58 partial Pokey sequences. The network was constructed using the NeighborNet algorithm. Sequences named CO_Scaffold_xxx were obtained from the v1.1 genome sequence of Daphnia pulex [ 36]. Colors represent species or hybrid state of isolates: red = D. pulex, light blue = D. arenata, grey = D. tenebrosa, black = European D. pulex, dark blue = European D. pulicaria, green = D. pulicaria, orange = putative pulex-pulicaria hybrids, purple = the isolate with D. tenebrosa mtDNA and a D. pulex nuclear genome. [file 1759-8753-4-7-S6.pdf]

**Additional File 6. Phylogenetic network of 58 partial *Pokey* sequences.**

The network was constructed using the NeighborNet algorithm. Sequences named CO_Scaffold_xxx were obtained from the v1.1 genome sequence of *Daphnia pulex* [36]. Colors represent species or hybrid state of isolates: red = *D. pulex*, light blue = *D. arenata*, grey = *D. tenebrosa*, black = European *D. pulex*, dark blue = European *D. pulicaria*, green = *D. pulicaria*, orange = putative *pulex-pulicaria* hybrids, purple = the isolate with *D. tenebrosa* mtDNA and a *D. pulex* nuclear genome.

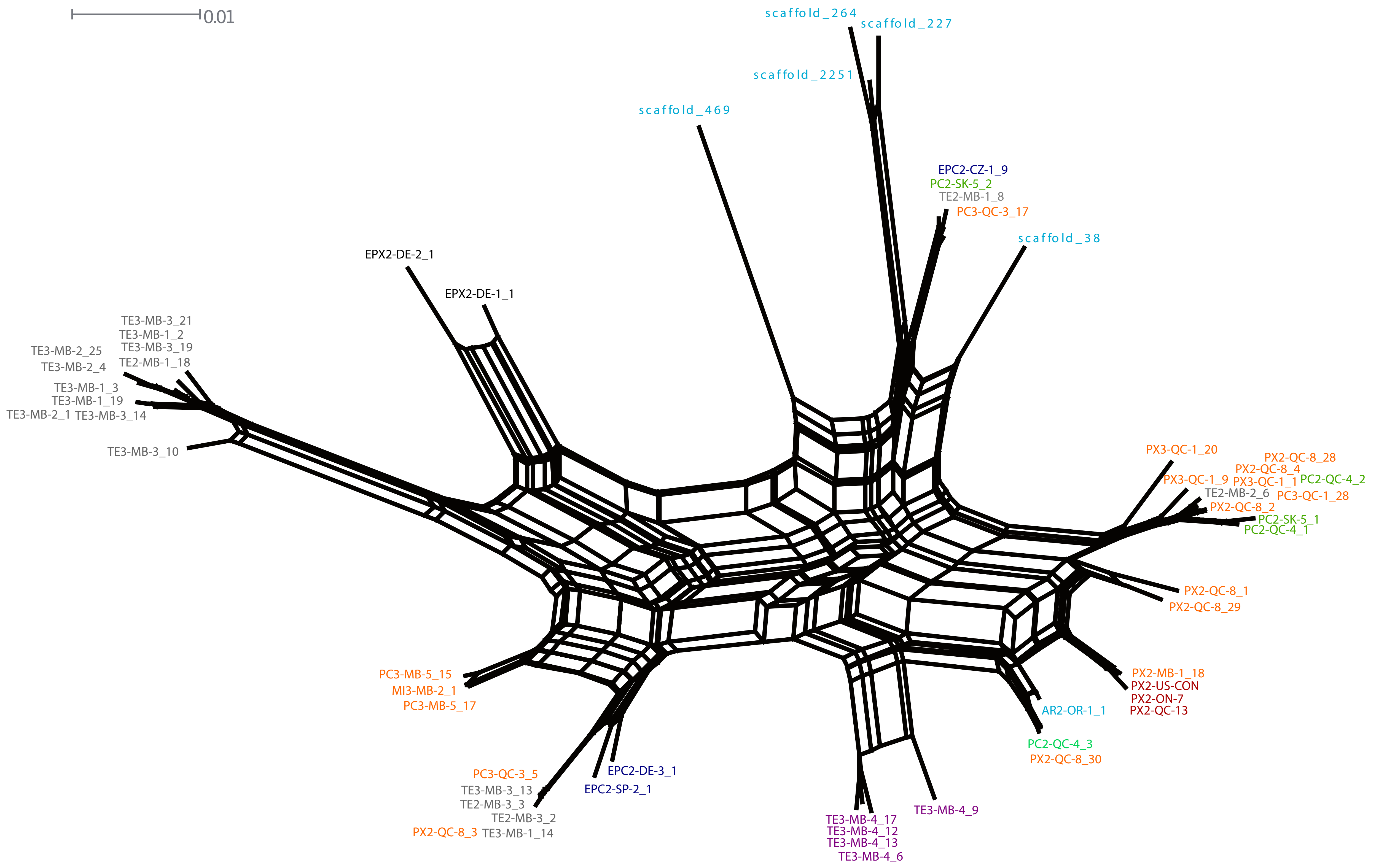
0.01
scaffold_264
scaffold_227
scaffold_2251
scaffold_469
EPC2-CZ-1_9
PC2-SK-5_2
TE2-MB-1_8
PC3-QC-3_17
scaffold_38
EPX2-DE-2_1
EPX2-DE-1_1
TE3-MB-3_21
TE3-MB-1_2
TE3-MB-3_19
TE3-MB-2_25
TE2-MB-1_18
TE3-MB-2_4
TE3-MB-1_3
TE3-MB-1_19
TE3-MB-2_1
TE3-MB-3_14
TE3-MB-3_10
PX3-QC-1_20
PX2-QC-8_28
PX2-QC-8_4
PX3-QC-1_9
PX3-QC-1_1
PC2-QC-4_2
TE2-MB-2_6
PC3-QC-1_28
PX2-QC-8_2
PC2-SK-5_1
PC2-QC-4_1
PX2-QC-8_1
PX2-QC-8_29
PC3-MB-5_15
MI3-MB-2_1
PC3-MB-5_17
PX2-MB-1_18
PX2-US-CON
PX2-ON-7
PX2-QC-13
AR2-OR-1_1
PC2-QC-4_3
PX2-QC-8_30
PC3-QC-3_5
EPC2-DE-3_1
TE3-MB-3_13
EPC2-SP-2_1
TE2-MB-3_3
TE2-MB-3_2
PX2-QC-8_3
TE3-MB-1_14
TE3-MB-4_9
TE3-MB-4_17
TE3-MB-4_12
TE3-MB-4_13
TE3-MB-4_6
