# Supplementary material for: Evolution of a transposon in Daphnia hybrid genomes
Source: Mob DNA. 2013 Feb 6;4:7. doi: 10.1186/1759-8753-4-7 (PMC3575242; doi:10.1186/1759-8753-4-7)
Supplement: Additional file 7 — Pokey RFLP haplotypes amplified from 41 isolates of the Daphnia pulex complex. Location codes are defined in Additional file 1. A one letter code allows differentiation of geographical regions in the same state or province: C = Churchill, MB, CAN; W = Winnipeg, MB, CAN; K = Kuujjuarapik, QC, CAN; M = Metis, QC, CAN; S = Sainte-Foy, QC, CAN. Taxonomic codes are as follows: EPC = European D. pulicaria; PC = D. pulicaria; PX = D. pulex; TE = D. tenebrosa; Hyb= pulex-pulicaria hybrid; Int = introgressed isolate. [file 1759-8753-4-7-S7.pdf]

Additional File 7. ***Pokey*** **RFLP haplotypes amplified from 41 isolates of the** ***Daphnia pulex*** **complex.**

Location codes are defined in Table 2. A one letter code allows differentiation of geographical regions in the same state or province: C = Churchill, MB, CAN; W = Winnipeg, MB, CAN; K = Kuujjuarapik, QC, CAN; M = Metis, QC, CAN; S = Sainte-Foy, QC, CAN. Taxonomic codes are as follows: EPC = European *D. pulicaria*; PC = *D. pulicaria*; PX = *D. pulex*; TE = *D. tenebrosa*; Hyb = pulex-pulicaria hybrid; Int = introgressed isolate.

Isolates	Locality	Taxonomy	Ecological niche	RFLP haplotypes							
				1	2	3	4	5	6	7	8
PX2-IL-1	IL, USA	PC	Lake		x						x
PX2-IL-2	IL, USA	PC	Lake		x						x
PX2-IL-3	IL, USA	PC	Lake		x						x
PX2-IL-4	IL, USA	PC	Lake		x						
PC2-IN-1	IN, USA	PC	Lake		x						
PC2-IN-2	IN, USA	PC	Lake		x						
PC2-MB-3	W, MB, CAN	PC	Lake								x
EPC2-CZ-1	CZR	EPC	Lake			x					
EPC2-SP-2	ESP	EPC	Lake				x				
PX2-ON-3	ON, CAN	PX	Pond	x							
PX2-ON-2	ON, CAN	Hyb	Pond	x							x
PX2-ON-10	ON, CAN	Hyb	Pond	x	x						
PX2-ON-4	ON, CAN	PX	Pond	x							
PX2-QC-5	S, QC, CAN	Hyb	Pond	x	x						
PX2-QC-6	S, QC, CAN	Hyb	Pond	x	x						
PX2-MI-7	MI, USA	Hyb	Pond	x		x	x			x	x
PX2-QC-9	M, QC, CAN	Hyb	Pond						x		
PX2-QC-12	M, QC, CAN	Hyb	Pond	x							
TE3-MB-1	C, MB, CAN	TE	Toundra Pond			x	x				
TE3-MB-2	C, MB, CAN	TE	Toundra Pond			x	x				
TE3-MB-3	C, MB, CAN	TE	Toundra Pond			x					
TE2-MB-1	C, MB, CAN	TE	Toundra Pond			x					x
TE2-MB-2	C, MB, CAN	TE	Toundra Pond		x						
TE2-MB-3	C, MB, CAN	TE	Toundra Pond				x				

PC3-QC-1	K, QC, CAN	Hyb	Toundra pond		x						
PC3-QC-2	K, QC, CANj	Hyb	Toundra pond		x	x					x
PC3-QC-7	K, QC, CAN	Hyb	Toundra pond						x		x
TE3-MB-4	C, MB, CAN	Int	Rock bluff		x			x			
PC3-MB-4	C, MB, CAN	Hyb	Rock bluff				x				
PC3-MB-5	C, MB, CAN	Hyb	Rock bluff				x				
PC3-MB-6	C, MB, CAN	Hyb	Rock bluff			x					
PX2-MB-3	C, MB, CAN	Hyb	Rock bluff	x							
MI3-MB-1	C, MB, CAN	Hyb	Rock bluff	x			x		x		
PX2-MB-1	C, MB, CAN	PX	Rock bluff				x				
MI3-SF-2	C, MB, CAN	Hyb	Rock bluff				x		x		
PX2-MB-2	C, MB, CAN	PX	Rock bluff			x	x				
PX2-QC-8	K, QC, CAN	Hyb	Rock bluff	x	x	x	x				x
PC3-QC-3	K, QC, CAN	Hyb	Rock bluff			x	x				
PX3-QC-1	K, QC, CAN	Hyb	Rock bluff	x	x						x
PX3-QC-2	K, QC, CAN	Hyb	Rock bluff	x							
PC2-QC-4	K, QC, CAN	PC	Rock bluff	x	x						
