# Supplementary material for: Evolution of a transposon in Daphnia hybrid genomes
Source: Mob DNA. 2013 Feb 6;4:7. doi: 10.1186/1759-8753-4-7 (PMC3575242; doi:10.1186/1759-8753-4-7)

Additional File 5. One possible ancestral *Pokey* recombination graph.

The graph was constructed from 40 different *Pokey* sequences from members of the *Daphnia pulex* complex and shows 89 recombination events. Ovals at the bottom of the graph represent *Pokey* alleles. Ovals with a trifurcation represent putative recombination events. Colors represent species or hybrid state of isolates: red = *D. pulex*, light blue = *D. arenata*, grey = *D. tenebrosa*, black = European *D. pulex*, dark blue = European *D. pulicaria*, green = *D. pulicaria*, orange = putative *pulex-pulicaria* hybrids, purple = the isolate with *D. tenebrosa* mtDNA and a *D. pulex* nuclear genome. Red lines represent the evolutionary history of the Pokey allele TE3-MB-4_12.

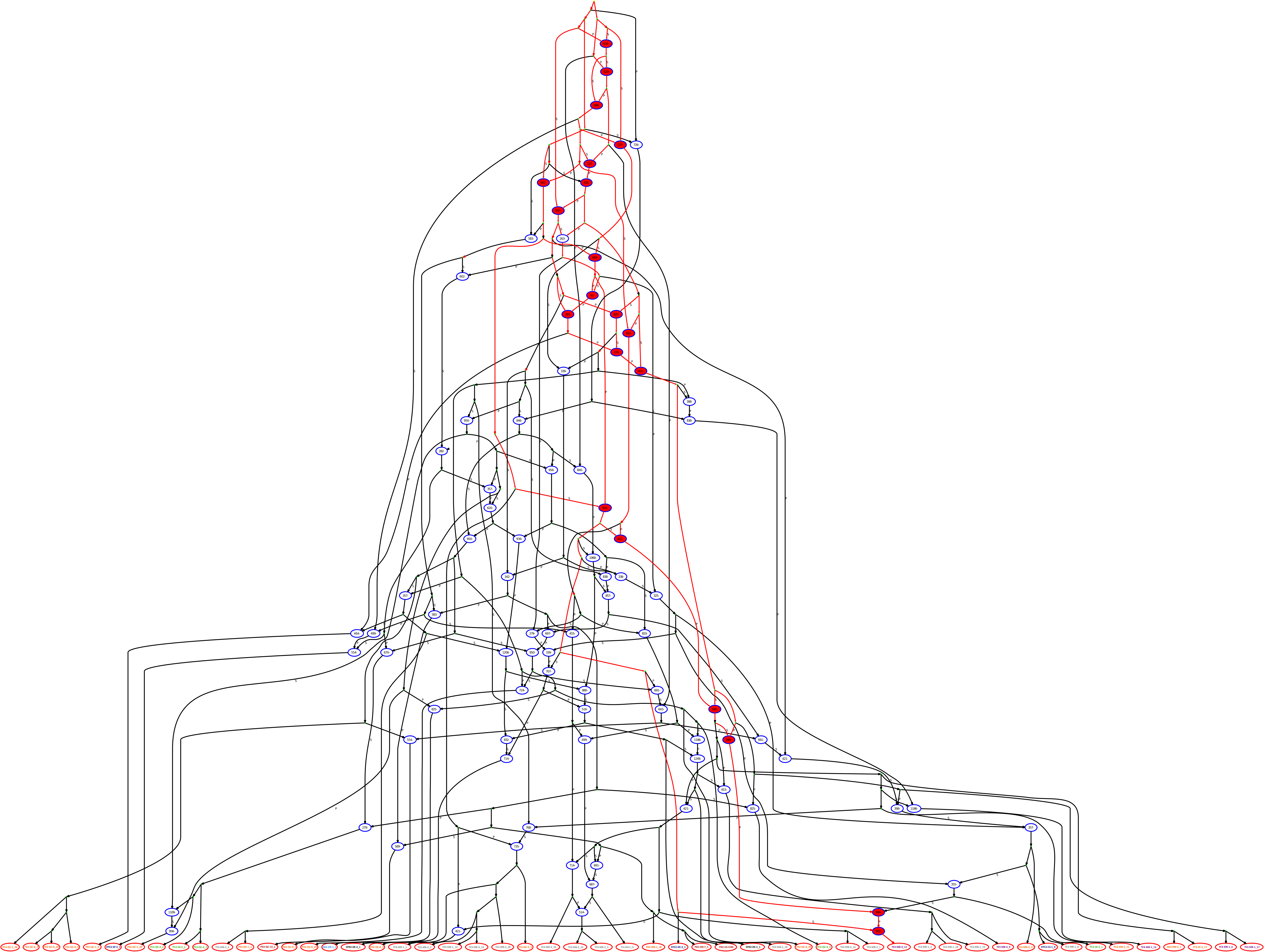

Supplement: Additional file 5 — One possible ancestral Pokey recombination graph. The graph was constructed from 40 different Pokey sequences from members of the Daphnia pulex complex and shows 89 recombination events. Ovals at the bottom of the graph represent Pokey alleles. Ovals with a trifurcation represent putative recombination events. Colors represent species or hybrid state of isolates: red = D. pulex, light blue = D. arenata, grey = D. tenebrosa, black = European D. pulex, dark blue = European D. pulicaria, green = D. pulicaria, orange = putative pulex-pulicaria hybrids, purple = the isolate with D. tenebrosa mtDNA and a D. pulex nuclear genome. Red lines represent the evolutionary history of the Pokey allele TE3-MB-4_12. [file 1759-8753-4-7-S5.pdf]
